# A new analysis tool for individual-level allele frequency for genomic studies

**DOI:** 10.1186/1471-2164-11-415

**Published:** 2010-07-05

**Authors:** Hsin-Chou Yang, Hsin-Chi Lin, Mei-Chu Huang, Ling-Hui Li, Wen-Harn Pan, Jer-Yuarn Wu, Yuan-Tsong Chen

**Affiliations:** 1 Institute of Statistical Science, Academia Sinica, Taipei 115, Taiwan; 2 Institute of Biomedical Sciences, Academia Sinica, Taipei 115, Taiwan

## Abstract

**Background:**

Allele frequency is one of the most important population indices and has been broadly applied to genetic/genomic studies. Estimation of allele frequency using genotypes is convenient but may lose data information and be sensitive to genotyping errors.

**Results:**

This study utilizes a unified intensity-measuring approach to estimating individual-level allele frequencies for 1,104 and 1,270 samples genotyped with the single-nucleotide-polymorphism arrays of the Affymetrix Human Mapping 100K and 500K Sets, respectively. Allele frequencies of all samples are estimated and adjusted by coefficients of preferential amplification/hybridization (CPA), and large ethnicity-specific and cross-ethnicity databases of CPA and allele frequency are established. The results show that using the CPA significantly improves the accuracy of allele frequency estimates; moreover, this paramount factor is insensitive to the time of data acquisition, effect of laboratory site, type of gene chip, and phenotypic status. Based on accurate allele frequency estimates, analytic methods based on individual-level allele frequencies are developed and successfully applied to discover genomic patterns of allele frequencies, detect chromosomal abnormalities, classify sample groups, identify outlier samples, and estimate the purity of tumor samples. The methods are packaged into a new analysis tool, ALOHA (**A**llele-frequency/**L**oss-**o**f-**h**eterozygosity/**A**llele-imbalance).

**Conclusions:**

This is the first time that these important genetic/genomic applications have been simultaneously conducted by the analyses of individual-level allele frequencies estimated by a unified intensity-measuring approach. We expect that additional practical applications for allele frequency analysis will be found. The developed databases and tools provide useful resources for human genome analysis via high-throughput single-nucleotide-polymorphism arrays. The ALOHA software was written in R and R GUI and can be downloaded at http://www.stat.sinica.edu.tw/hsinchou/genetics/aloha/ALOHA.htm.

## Background

Allele frequency denotes the relative frequency of an allele compared with the total frequency of all alleles at a marker locus. It is one of the most important population indices and has been broadly applied to genetic/genomic research [1-5]. The generalized concept of allele frequency has two aspects: individual-level allele frequency and population-level allele frequency. The former represents a within-individual relative frequency of alleles and its standard error reflects inter-cell variability in an individual; the latter represents a within-population relative frequency of alleles and its standard error reflects inter-individual variability in a population [6].

Using the most abundant genetic marker in the human genome, namely the single-nucleotide polymorphism (SNP), both individual-level and population-level allele frequencies can be estimated using a (genotype-based) allele-counting approach and an (intensity-based) intensity-measuring approach (**Appendix A**). This paper focuses on the intensity-measuring allele frequency because this allele frequency estimate is insensitivity to genotyping errors and preserves data information that might be lost in conventional genotype-based analyses. For instance, the genomic abnormality of a particular sample, for instance NA18996 from the Japanese in population in the International HapMap Project [7-10], can be easily observed by individual-level allele frequencies using an intensity-measuring approach [Additional file 1, **Supplemental Figure S1 (A)**] but not by using an allele-counting approach [Additional file 1, **Supplemental Figure S1 (B)**]. The finding motivated us to analyze intensity-based allele frequencies for extraction of information lost in conventional genotype-based analyses.

An intensity-measuring approach can accurately estimate allele frequencies with the aid of an adjustment for preferential amplification/hybridization [11]. The coefficient of preferential amplification/hybridization (CPA) is used to quantify preferential amplification/hybridization and reduce the estimation bias of allele frequency [12-19]. Consider a SNP with genotype *AA*, *Aa *or *aa*. In comparison with an unadjusted individual-level allele frequency [Additional file 2, **Supplemental Figure S2 (A)**], a CPA-adjusted frequency of allele *A *moves toward the expected values of 1, 0.5 and 0 for SNPs with genotypes *AA*, *Aa *and *aa*, respectively [Additional file 2, **Supplemental Figure S2 (B)**]. In comparison with an unadjusted population-level allele frequency from a DNA pool of 240 individuals [Additional file 3, **Supplemental Figure S3 (A)**], a CPA-adjusted frequency of allele *A *moves toward the true allele frequencies obtained from an individual genotyping experiment [Additional file 3, **Supplemental Figure S3 (B)**] [6]. The results demonstrate the important role of CPA adjustment in allele frequency estimation, thereby expanding applications of allele frequency in various genomic science disciplines. The features of CPA have been studied, but not exhaustively [14,15,17]. Therefore, we undertook an in-depth investigation of the relationship between CPA and important factors including sample size, time of data acquisition, effects of laboratory site, type of gene chip, ethnicity effects, and phenotypic status, and we constructed new public CPA and allele frequency databases.

Population-level allele frequency has many important applications and has been well discussed [1-4]. This paper mainly focuses on individual-level allele frequency and investigates its applications in genetic/genomic studies, including the discovery of allele frequency patterns, identification of chromosomal aberrations (including aneuploidy, loss of heterozygosity (LOH), and allelic imbalance (AI)), outlier detection, and sample classification (including stratification by population and gender). This is the first time that these important genetic/genomic applications have been simultaneously conducted based on individual-level allele frequencies estimated by a unified intensity-measuring approach.

## Methods

### Samples

This study analyzes genotype and intensity data from several large genomic projects. The first dataset consists of 367 and 448 Taiwanese samples genotyped using the Affymetrix Human Mapping 100K Set and 500K Set (Affymetrix, San Diego, CA, USA), respectively, from the Taiwan Han Chinese Cell and Genome Bank [20]. The second dataset consists of 175 and 198 hypertension patients genotyped using the Affymetrix Human Mapping 100K Set and 500K Set, respectively, from the Academia Sinica Multi-Centered Hypertension Genetic Study. The third dataset consists of 30 African trios of Yoruba in Ibadan (YRI), 30 Caucasian trios of residents in CEPH Utah (CEU), and 90 Asians (45 Han Chinese in Beijing (CHB) and 45 Japanese in Tokyo (JPT)) from the International HapMap Project [7-10], where all 270 samples were genotyped using both the Affymetrix Human Mapping 100K Set and 500K Set. The fourth dataset consists of 242 and 304 leukaemia cancer patients who were genotyped using the Affymetrix Human Mapping 100K Set and 500K Set, respectively; in addition, 50 normal controls were genotyped with the Affymetrix Human Mapping 100K and 500K Sets from an acute lymphoblastic leukaemia (ALL) project [21,22]. All participants involved in the genomic projects signed Informed Consent Forms.

### Genotyping

All samples were genotyped with the Affymetrix Human Mapping 100K Set and/or 500K Set (Affymetrix, San Diego, CA, USA), which contain 116,204 SNPs with a median inter-marker distance of 8.5 kb and 500,568 SNPs with a median inter-marker distance of 2.5 kb, respectively. The data sheets and genotyping manuals for the two SNP chips can be downloaded at http://www.affymetrix.com/. For details of the SNP genotyping experiments refer to Yang *et al*. [23] for the Taiwanese normal samples and hypertensive patients, The International HapMap Consortium [7-10] for the International HapMap Project samples, and Mullighan *et al*. [21,22] for the ALL Project samples. SNP genotype calling algorithms, DM (Dynamic Model) [24] and BRLMM (Bayesian Robust Linear Model with Mahalanobis Distance Classifier) [25], were used for the Affymetrix Human Mapping 100K Set and 500K Set, respectively.

### Allele frequency estimation

An individual-level allele frequency is the proportion of a specific allele in a genotype. Here we formulate the existing procedures of allele frequency estimation. Consider an example of a SNP with genotype *aa*, *Aa *or *AA*. Two methods can be used to estimate an individual-level allele frequency. First, the allele-counting approach uses genotype data from an individual genotyping experiment (IGE). The number of allele *A *is counted and then used to calculate the proportion of allele *A *in a genotype. Besides no calls, there are only three possible outcomes of an allele frequency estimate for a SNP. Therefore, allele frequency estimates are 0, 0.5 and 1, corresponding to genotypes *aa*, *Aa *and *AA*, respectively (**Equation (A1)**). Second, the intensity-measuring approach uses intensity data from an IGE. The frequency of allele *A *is estimated by calculating the ratio of intensities pertaining to allele *A *relative to the total intensity of two alleles, where intensities are adjusted by considering a CPA. The ratio reflects a relative amount of allele *A *compared with the total amount of two alleles at a SNP for an individual (**Equation (A2)**).

In contrast to an individual-level allele frequency, a population-level allele frequency is the proportion of a specific allele in a study population. The allele frequencies can be estimated based on data from an IGE or a pooled allelotyping experiment (PAE). In an IGE, individual-level allele frequencies are estimated by using genotype data or intensity data as mentioned above. Then a population-level allele frequency is estimated by taking an average over individual-level allele frequencies from genotype data (**Equation (A3)**) or intensity data (**Equation (A4)**). In a PAE, intensity data are available, but individual genotype data are not. A relative intensity of allele *A *in a DNA pool, which is constructed by mixing genomic DNA from multiple samples, is calculated to estimate a population-level allele frequency (**Equation (A5)**). The detailed procedures for allele frequency estimation and CPA adjustment are described in **Appendix A**.

### Identification of chromosomal aberrations

We develop multiple chromosomal aberration indices and a sliding-window approach in concert with a standard individual-level allele frequency plot to identify chromosomal aberrations such as aneuploidy, AI, LOH, long-contiguous-stretch-of-homozygosity (LCSH), and so on. For an individual and a SNP, individual-level allele frequency is compared with genotype-specific reference confidence intervals. Index  is used to detect AI genomic segments characterized by SNP points with allele frequencies outside the allele frequency confidence intervals of three genotypes. Index  is used to detect LOH or LCSH, genomic regions characterized by SNP points with contiguous homozygous calls (**Appendix B**). The two indices are used to identify unusual SNPs point by point. Furthermore, based on all patients and normal controls, two multipoint indices, (**Equation (B1)**) and (**Equation (B2)**) are calculated in each window using a sliding-window approach. In addition, the smoothed indices,  and , are also calculated using a spline smoothing technique. We identify chromosomal aberrations by pinpointing the genomic regions where the indices of a patient are higher than the 95%-quantile indices of normal controls. Detailed procedures for identifying chromosomal aberrations are described in **Appendix B**.

### Sample classification and outlier detection

We apply an allele frequency biplot based on individual-level allele frequencies to classify samples and detect outliers. An allele frequency biplot, which uses a singular value decomposition to decompose an allele frequency matrix into a sample matrix and a SNP matrix (**Equations (C1) and (C2)**), projects samples and SNPs onto a two-dimensional plane simultaneously. The first dimension is a sample coordinate constructed by the first two columns of the sample matrix, and the second dimension is a SNP coordinate constructed by the first two columns of the SNP matrix. The rank-2 biplot configuration can be utilized for sample classification, outlier detection and SNP clustering. First, sample classification allows for samples with a similar allele frequency distribution to be clustered and used to study population stratification and gender grouping. Second, outlier detection identifies samples far away from the majority of samples for a further examination prior to downstream analyses. Third, SNP clustering identifies a collection of SNPs physically close to specific sample groups. The SNP patterns are used to separate sample groups and explain sample characteristics. Detailed procedures for constructing an allele frequency biplot are described in **Appendix C**.

## Results

### Coefficient of preferential amplification/hybridization

We characterize genomic patterns of CPA based on the three genome projects, the Taiwan Han Chinese Cell and Genome Bank [20], the Academia Sinica Multi-Center Hypertension Genetic Study, and the International HapMap Project [7-10]. Both Affymetrix 100K and 500K Sets are considered. This work extends our previous study by using richer study samples and denser SNP chips [17]. We calculate CPA based on an unbiased estimator [12] and construct public CPA databases for the Taiwanese population, ethnic-specific populations (African, Asian, and Caucasian populations), and a cross-ethnicity group (a combination of African, Asian and Caucasian populations). The CPA databases are available online at http://140.109.72.48/index.htm. Three CPA query methods (keyword query, general query and advanced query) are provided. In addition, we examine the relationship between CPA and important factors including: (1) sample size (45, 90, 180, and 367 individuals); (2) time of data acquisition (four genotyping periods) and effects of laboratory site (two sites); (3) gene chip type (Affymetrix Human Mapping 100K Set and 500K Set); (4) ethnicity effects (Africans, Asians, and Caucasians); and (5) phenotypic status (normal controls and young-onset hypertension patients). Results of the investigation of these issues are summarized in order they are presented here.

First, CPA can be estimated well for a moderate sample size [Additional file 4, **Supplemental Figure S4 (A)**], but its variance decreases significantly with increasing sample size [Additional file 4, **Supplemental Figure S4 (B)**]. Based on a total of 367 Taiwanese samples that were genotyped with the Affymetrix Human Mapping 100K Set, 45, 90 and 180 samples are randomly selected from the 367 samples. Pairwise correlation coefficients of log_2_(CPA) obtained from different sample sizes are calculated, and the lowest correlation coefficient is greater than 0.975. In the fitted quadratic regression, an intercept term is close to 0 (i.e., the regression curve passes through the origin), and the regression coefficients of the linear term and quadratic term are close to 1 and 0, respectively. In other words, CPAs are close even when they are calculated from a relatively small sample size, implying that CPA can be estimated well even for a moderate sample size [Additional file 4, **Supplemental Figure S4 (A)**]. Note that, with respect to each SNP, only heterozygous individuals have been used to estimate CPA. The expected number of heterozygous individuals is the number of total samples multiplied by the probability that a SNP is heterozygous. CPA variances pertaining to the four sample sizes are calculated. The overall SNP averages of ratios of CPA variances of 180 versus 367 samples (green points), 90 versus 367 samples (red points), and 45 versus 367 samples (blue points) are 1.42, 1.99, and 3.90, respectively. The larger the sample size, the smaller the variability of CPA [Additional file 4, **Supplemental Figure S4 (B)**].

Second, CPA is insensitive to data from different genotyping time periods [Additional file 5, **Supplemental Figure S5 (A)**] and different laboratories [Additional file 5, **Supplemental Figure S5 (B)**]. To evaluate time effect on CPA, CPAs are calculated based on data from the same genotyping laboratory but from four time periods of data acquisition (2005/05/04, 2006/01/09, 2006/03/17 and 2006/06/29), and sample sizes in experiments are close (95, 96, 90 and 76). All the pairwise correlation coefficients of log_2_(CPA) are greater than 0.966, and quadratic regression curves act like a linear regression line passing through the origin and having a slope of 1, implying that CPA is robust to data from different genotyping periods [Additional file 5, **Supplemental Figure S5 (A)**]. We also examine the effect of genotyping sites on CPA. CPAs are calculated based on data from the 90 Asian samples in the International HapMap Project and 95 Taiwanese samples. These two datasets with similar sample sizes were collected from Asian populations but genotyped at different times and sites. A high correlation of CPA between the two datasets is found across chromosomes, implying that CPA is robust to data from different genotyping sites [Additional file 5, **Supplemental Figure S5 (B)**].

Third, CPAs from the Affymetrix Human Mapping 100K and 500K Sets yield similar genomic patterns [Additional file 6, **Supplemental Figures S6 (A) - (D)**]. Means (standard deviations) of log_2_(CPA) for 100K and 500K Sets are 0.04 (0.54) and 0.05 (0.70), respectively. The majority of log_2_(CPA) are bounded by ±1. Moreover, we examine genome-wide distributions of CPA for different ethnic groups (CHB, JPT, YRI, and CEU), Taiwanese samples and their combination) and different SNP chips (Affymetrix 100K and 500K Sets). The results show that genome-wide CPA can be well modeled by using log-normal distributions, where the results of Taiwanese samples are shown [Additional file 7, **Supplemental Figures S7 (A) and (B)**]. The finding is useful for discussions of theoretical sampling distribution of CPA-adjusted allele frequency estimates and statistical tests.

Fourth, genomic distributions of CPA between ethnic groups may differ [Additional file 8, **Supplemental Figures S8 (A) and (B)**]. CPAs are calculated based on data from different populations, including 45 Han Chinese (CHB), 45 Japanese (JPT), 60 Africans (YRI), 60 Caucasians (CEU), 90 Asians (CHB+JPT), and 210 combined samples (CHB+JPT+YRI+CEU). Results show that correlation of CPAs among populations is dependent on the ethnic populations selected [Additional file 8, **Supplemental Figure S8 (A)**]. Among all pair-wise comparisons, two Asian populations, CHB and JPT, have the highest correlation (r = 0.966), depicted by a flat ellipse; the two Asian populations and Caucasian populations have the lowest correlation (r = 0.845 for CHB and CEU and r = 0.845 for JPT and CEU), depicted by round ellipses. Note that comparison of the Caucasian and the combined Asian populations present a nephroid ellipse, where the 95% confidence interval of the mean regression curve does not pass through the origin, and the slope term deviates from unity. This suggests a relatively large discrepancy of CPAs between Asian and Caucasian populations. CPAs of the African population are located in an intermediate position between the Asian and Caucasian populations, and the order is shown in a tree diagram [Additional file 8, **Supplemental Figure S8 (B)**].

Finally, CPA distributions between hypertension patients and normal controls are similar [Additional file 9, **Supplemental Figure S9**]. CPAs are calculated based on a total of 180 Taiwanese normal control samples and 175 Taiwanese hypertension patient samples that were genotyped with the Affymetrix Human Mapping 100K Set in the same laboratory. A high correlation (r = 0.980) is observed. This result implies that the pooled DNA association mapping of a Taiwanese hypertension study may use the same CPA for case and control groups.

### Allele frequency

**Figure 1:**
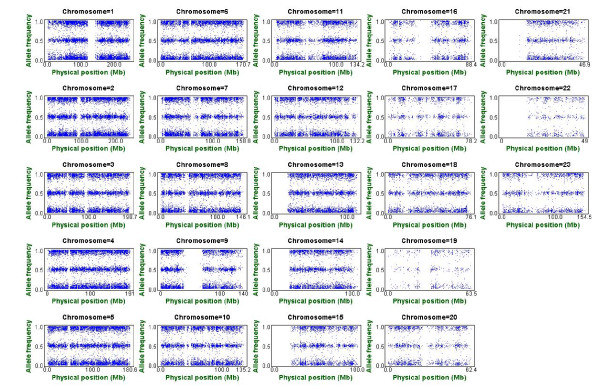
**Allele frequency plot of a normal sample (NA19206) from the YRI population based on the Affymetrix Human Mapping 100K Set**. This figure consists of 23 subfigures. Each subfigure presents an allele frequency plot for one chromosome. The vertical axis is the estimated individual-level allele frequency, and the horizontal axis is physical position (Mb). Each point denotes a SNP, and the gap in each subplot represents the centromeric gap. Individual-level allele frequencies of SNPs on each chromosome were close to the expected values of 0, 0.5 and 1.

Based on our CPA databases, we estimate individual-level allele frequencies using the intensity-measuring approach for samples from different populations, including (1) Affymetrix Human Mapping 100K Set: 367 Taiwanese samples and 270 HapMap samples, and (2) Affymetrix Human Mapping 500K Set: 448 Taiwanese samples and 270 HapMap samples. The pattern of allele frequency can be observed in a standard allele frequency plot. For example, for a normal female sample (NA19206) from the YRI population in the International HapMap Project (The International HapMap Consortium, 2003-2007), with the exception of some noisy SNP points, an allele frequency plot exhibits three bands formed by SNPs with an individual-level allele frequency close to 0, 0.5 or 1 (Figure 1 and **Equation (A2)**). Allele frequency plots of cancer patients with chromosomal aberrations will be shown later in Identification of Chromosomal Aberrations. In addition, we establish individual-level allele frequency databases that provide SNP annotation, allele frequencies and the summary statistics for the HapMap samples for both the Affymetrix Human Mapping 100K and 500K Sets. The databases are available online at http://140.109.72.48/index.htm. Three allele frequency query methods (keyword query, general query and advanced query) are provided. The individual-level allele frequency estimates and databases are applied to genomic pattern detection, chromosomal aberration analysis, classification analysis, and outlier detection.

### Identification of chromosomal aberrations

**Figure 2:**
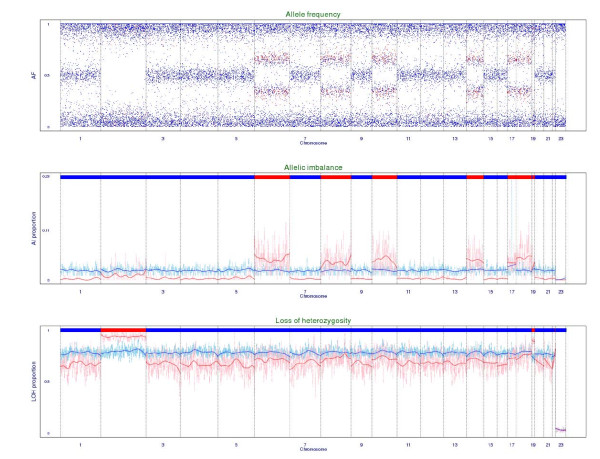
**Chromosomal aberration plots of a hyperdiploidy leukaemia patient based on the Affymetrix Human Mapping 100K Set**. A chromosomal aberration plot consists of an extended allele frequency plot, an allelic imbalance (AI) plot, and a loss of heterozygosity (LOH) plot from the top to the bottom (**Appendix B**). In the extended allele frequency plot, a lot of the AI SNPs (red points) were found on whole chromosomes 6, 8, 10, 14, 17, 18 and 19. In the AI plot, a whole-chromosome region of AI (red bar in the top panel) was found on chromosomes 6, 8, 10, 14, 17, 18 and 19. In the LOH plot, a whole-chromosome region of LOH (red bar in the top panel) was found on chromosomes 2 and 19.

**Figure 3:**
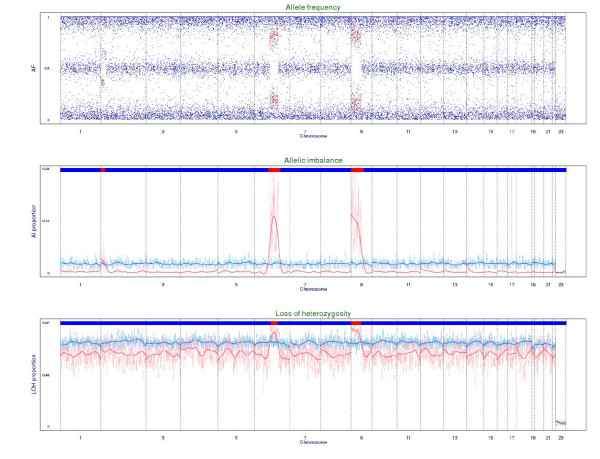
**Chromosomal aberration plots of a T-ALL patient based on the Affymetrix Human Mapping 100K Set**. A chromosomal aberration plot consists of an extended allele frequency plot, an allelic imbalance (AI) plot, and a loss-of-heterozygosity (LOH) plot from the top to the bottom (**Appendix B**). In the extended allele frequency plot, clusters of AI SNPs (red points) were found on chromosomal regions of 2p, 6q and 9p. The regions of 2p, 6q and 9p were also identified in the AI plot (red bar in the top panel). The regions of 6q and 9p were also identified in the LOH plot (red bar in the top panel).

Individual-level allele frequency can be used to identify chromosomal aberrations, such as aneuploidy, LOH, AI, and so on, which contribute to the complex genomic profiles of many cancer patients. Based on our constructed databases of CPA and individual-level allele frequency, we apply the developed sliding-window LOH and AI detectors to identify chromosomal aberrations for the 242 and 304 ALL patients that were genotyped with the Affymetrix Human Mapping 100K Set and 500K Set, respectively, in the ALL Project. Two examples are given here to demonstrate whole-chromosome aberration and segment aberration. The first example is a male leukaemia patient with hyperdiploidy (Hyperdip50-SNP-#27). His genome exhibits AI on chromosomes 6, 8, 10, 14, 17, and 18 and LOH on chromosomes 2, 19 and 22 (Figure 2 and **Appendix B**). The second example is a male T-cell ALL patient (T-ALL-SNP-#49). Three chromosomal regions, 2p, 6q, and 9p, of AI are identified, with chromosomes 6q and 9p also show LOH (Figure 3 and **Appendix B**).

High proportions of LOH and AI are found in the ALL study. In the analysis of 242 patients genotyped with the Affymetrix 100K Set, 215 samples (88.80%) with AI are found, and 93 of these (43.26%) have whole-chromosome AI. Among the 242 patients, 154 samples (63.63%) with LOH are found, and 26 of these (16.88%) have whole-chromosome LOH. In the analysis of 304 patients genotyped with the Affymetrix 500K Set, 261 samples (85.86%) with AI are found, and 53 of these (20.31%) have whole-chromosome AI. Among the 304 patients, 240 samples (78.95%) with LOH are found, and 35 of these (14.58%) have whole-chromosome LOH.

### Group classification and outlier detection

Based on individual-level allele frequencies, an allele frequency biplot is developed to classify samples belonging to different ethnic groups and gender groups and identify outlier samples.

**Figure 4:**
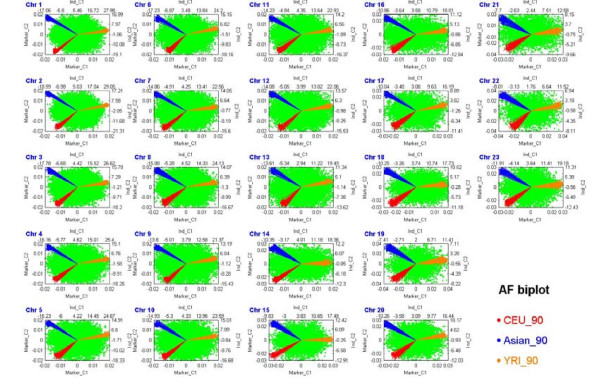
**Allele frequency biplots of the three populations in the International HapMap Project dataset based on the Affymetrix Human Mapping 500K Set**. A genome-wide allele frequency biplot of data for 90 CEU, 90 YRI, and 90 subjects of Asian descent (45 CHB and 45 JPT). The figure consists of 23 subfigures. Each subfigure presents an allele frequency biplot of one chromosome, where the four axes reflect the first two individual components and the first two SNP components in a rank-2 biplot (**Appendix C**). Samples from YRI (yellow line), CEU (red line) and Asian (blue line) were clearly classified into three groups.

**Figure 5:**
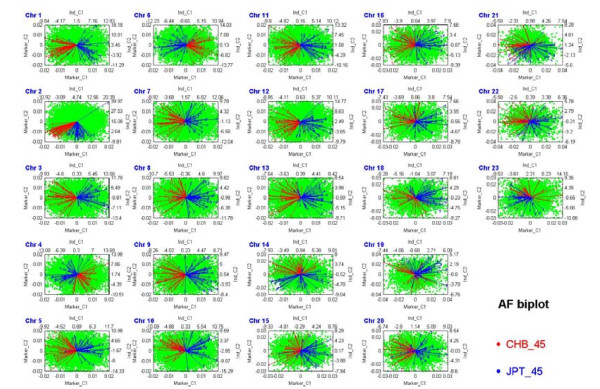
**Allele frequency biplots of the Asian population in the International HapMap Project dataset based on the Affymetrix Human Mapping 500K Set**. A genome-wide allele frequency biplot of data for 90 subjects of Asian descent (45 CHB and 45 JPT). The figure consists of 23 subfigures. Each subfigure presents an allele frequency biplot of one chromosome, where the four axes reflect the first two individual components and the first two SNP components in a rank-2 biplot (**Appendix C**). Samples from CHB (red line) and JPT (blue line) are accurately classified as two distinct groups.

First, in an unsupervised cluster analysis of the International HapMap Project data, the majority of samples from African (YRI), Caucasian (CEU) and Asian (CHB and JPT) populations are clearly classified into three groups in an allele frequency biplot (Figure 4 and **Appendix C**). Furthermore, samples from Chinese (CHB) and Japanese (JPT) populations are also accurately classified as two distinct groups (Figure 5 and **Appendix C**). The results illustrate that individual-level allele frequencies are not only useful to distinguish the genetic differences of the Asian, Caucasian and African populations, but also able to distinguish the small differences between Asian subpopulations.

Second, allele frequencies can be used to judge the gender of a person from which a sample was obtained. Genetically, a female has two X chromosomes and a male has only one X chromosome. High homozygosity on the X chromosome should generally be observed in a male sample [Additional file 2, **Supplemental Figure S2 (B) **for a male and Figure 1 for a female]. Our analysis shows that an allele frequency biplot for the X chromosome can classify the gender of samples donors [Additional file 10, **Supplemental Figure S10**].

Third, allele frequency biplots can be used to detect outliers. For example, a JPT sample (NA1902) is some distance away from all other samples in a biplot of chromosome 2 (Figure 5) because this sample has a LCSH on this chromosome.

## Discussion

We apply individual-level allele frequencies, estimated using an intensity-measuring approach, to genetic and genomic studies. Under the framework, statistical inferences are drawn based on intensity data instead of genotype data. Compared with a genotype-based analysis that estimates allele frequencies via an allele-counting approach, our method has three advantages. First, the method can analyze data from DNA samples reflecting clonal heterogeneity and containing DNA from contaminating "normal" cells, which is often the case in cancer studies. Second, the method can be used to analyze data from mixed DNA of multiple samples for cost savings. Third, the method is insensitive to genotype calling errors, which frequently occur in aberrant chromosomal regions, such as segments with aneuploidy, LOH, AI, and so on. The performance of the methods developed for amplification/hybridization calibration, allele frequency estimation, group classification, outlier detection, and genomic aberration identification are examined comprehensively based on the data from large-scale genomic projects.

The proposed methods are not restricted to the Affymetrix GeneChip, but also are adapted to the Illumina BeadChip. For example, the individual-level allele frequency plot of a normal sample genotyped with the Illumina HumanHap550-Duo BeadChip also yields the expected three-band pattern similar to what is obtained with the Affymetrix GeneChip [Additional file 11, **Supplemental Figure S11**]. In addition to applications for individual-level allele frequency, the proposed intensity-measuring approach can be easily applied to study population-level allele frequency. For example, in a pooled allelotyping experiment [2,26-31], individual genotype information is not available, thereby the allele-counting approach fails to be applied; the proposed intensity-measuring approach, however, works well in this type of experiment (**Appendix A**). Allele frequency estimates of the pooled allelotyping experiment of a pool size of 240 are quite accurate compared to the true answers obtained from the individual genotyping experiment of all 240 samples (r = 0.983) [Additional file 3, **Supplemental Figure S3 (B)**]. Pooled DNA is constructed by mixing DNA from multiple individuals; therefore, allelotyping of pooled DNA provides a cost-saving alternative to an individual genotyping experiment and has been recognized as an efficient tool for identification of polymorphisms and mutations, homozygosity mapping, genetic association, and so on.

We have constructed large ethnicity-specific and cross-ethnicity CPA databases. Compared with our previous database [17], the new databases are improved by increased sample size and the addition of a dense SNP chip platform. Researchers may use our free database, thereby curtailing study costs. The characteristics of CPA extracted from our databases are summarized as follows. First, CPA variance decreases significantly as sample size increases; thus, a CPA database should be based on a large number of samples. Second, CPA is independent of the time and laboratory where the genotyping took place, and therefore the CPAs provided in our databases are applicable to studies carried out at different times and places. Third, the 100K and 500K gene chips yield similar CPA patterns. Fourth, discrepancies of CPAs in ethnic groups discourage the direct use of CPA from different ethnic groups. Fifth, most SNPs have a strongly positively correlated genomic distribution of CPA between phenotypic groups of hypertension. Sixth, log-normal distributions capture the genomic distributions of CPA well, and CPA is associated with GC content and genotype. These findings broaden our understanding of preferential amplification/hybridization and will benefit a wide variety of genetic/genomic studies. In addition, we also constructed a database of individual-level allele frequency, which provides useful materials for genetic/genomic research discussed in this paper.

Interestingly, when compared to the result of DNA copy number changes in T-ALL-SNP-#49 reported by Mullighan *et al*. [21], AI of chromosome 2p correlates well with amplification, whereas AI and LOH of chromosomes 6q and 9p are the result of deletions. These results demonstrate the feasibility of applying individual-level allele frequencies to identify chromosomal aberrations. Our method can be jointly used with some of the existing copy number analysis tools such as LB [32], PennCNV [33], QuantiSNP [34], dCHIP [35], and GenoCN [36] to move toward a more complete understanding of the mechanisms underlying chromosomal abnormalities.

In addition to identification of chromosomal aberrations, individual-level allele frequency can also be used to identify an occurrence of cell mixture. JPT sample NA18996 is an example with "abnormal" genomic patterns [Additional file 1, **Supplemental Figure S1 (A)**] that obviously deviate from the three-band pattern of a normal sample [Additional file 2, **Supplemental Figure S2 (B)**]. The individual-level allele frequency plot of sample NA18996 shows that the majority of this individual's genome have five allele frequency bands with the exception that some autosomal regions have two or three bands and the X chromosome has four bands, indicating that this sample may reflect a mixture of DNA samples. In addition, we show that individual-level allele frequency can also be used to estimate the level of contaminating "normal" cells (**Appendix D**). In the example of T-ALL-SNP-#49, the average allele frequencies (*f*_1_) of the abnormal regions of 6q and 9p were as follows: *f*_1 _= 0.192 for 6q, and *f*_1 _= 0.190 for 9p, implying an estimated range of contaminating "normal" cells of 0.235 - 0.250, which is close to the reported sample purity (blast percentage = 76%) reported by Mullighan *et al*. [21].

Our analyses show that individual-level allele frequencies and allele frequency biplots are useful for distinguishing genetic differences among ethnic populations. On the one hand, the method can be applied to examine population stratification, which is one of the most common factors underlying false positives in case-control association studies, through the identification of subgroups. On the other hand, the method is potentially useful to assign ethnicity for individuals with unclear ancestry information by superimposing a new sample point to the established ethnic groups using an allele frequency biplot, or to assign unknown forensic individuals to pre-study groups. Compared with a principal component analysis that can also be applied to study sample classification, an allele frequency biplot provides a more detailed visual appraisal of the relationship between SNPs and samples (**Appendix C**).

## Conclusion

Allele frequency has been studied for many years. With the aid of whole-genome SNP chip technology, high-throughput SNP data have become available. Based on the individual-level allele frequencies obtained from the unified estimation procedure, we developed new methods/software/databases to extract hidden genetic information and broaden the potential application of allele frequency to genomic research. Our new analysis tool, ALOHA (**A**llele-frequency/**L**oss-**o**f-**h**eterozygosity/**A**llele-imbalance), written in R and R GUI, provides for genome-wide analysis of allele frequency and detection of both LOH and AI. An allelefrequency biplot is also provided for sample classification, outlier detection, and SNP clustering. The software can be downloaded at http://www.stat.sinica.edu.tw/hsinchou/genetics/aloha/ALOHA.htm. New allele frequency and CPA databases have been created. Functions for data query modes (keyword query, general query, and advanced query) and data downloads are provided. The databases are available at http://140.109.72.48/index.htm. We expect that additional practical applications of allele frequency will be found and allele frequency analysis will continue to play a key role in genetic/genomic research.

## Abbreviations

AI: Allelic imbalance; ALL: Acute lymphoblastic leukaemia; ALOHA: Allele-frequency/Loss-of-heterozygosity/Allele-imbalance; CEU: CEPH Utah residents; CHB: Han Chinese in Beijing; CPA: Coefficient of preferential amplification/hybridization; IGE: Individual genotyping experiment; JPT: Japanese in Tokyo; LCSH: Long contiguous stretch of homozygosity; LOH: Loss of heterozygosity; PAE: Pooled allelotyping experiment; SNP: Single nucleotide polymorphism; YRI: Yoruba in Ibadan.

## Authors' contributions

HCY conceived of the study, developed statistical methods and prepared the manuscript. HCL and MCH programmed ALOHA software and analyzed the data with HCY. LHL contributed to discussion. WHP, JYW and YTC provided genetic data of Taiwanese samples. All authors read and approved the final manuscript.

## Appendices

### Appendix A. Allele frequency estimation

We summarize the estimation procedures for two types of allele frequencies, individual-level and population-level allele frequency, in this appendix. This summary focuses on the most abundant genetic marker, the SNP, in the human genome. SNP data can be generated from two types of SNP typing experiments, namely an individual genotyping experiment (IGE) and a pooled allelotyping experiment (PAE), which can be carried out using a customized platform (e.g., MALDI-TOF mass spectrometry) or a genome-wide platform (e.g., Affymetrix and Illumina SNP chips).

An IGE collects intensity data (e.g., mass spectrometry peak intensity provided by MALDI-TOF mass spectrometry or hybridization intensity provided by Affymetrix SNP chips) and then uses a SNP calling algorithm to assign genotype calls to SNPs based on intensity data. Both individual-level and population-level allele frequencies can be estimated by using an allele-counting approach based on genotypic data and an intensity-measuring approach based on intensity data.

In contrast to an IGE, which analyzes DNA from each individual, a PAE analyzes mixed DNA from multiple individuals. With PAE it is difficult to extract genotype information for individuals although the same genotyping platform is employed for PAE as IGE. A PAE collects intensity data but cannot determine individual genotypes, and is therefore unable to estimate individual-level allele frequency; nevertheless, a PAE provides a cost-efficient way to estimate population-level allele frequency by using an intensity-measuring approach.

We introduce the estimating procedures as follows. Suppose that there are *n *independent individuals in total. Consider a SNP with genotype *AA*, *Aa *or *aa*. Let (*p*_*i*_,*i *= 1,...,*n*) denote individual-level allele frequencies for *n *individuals and *p *denote a population-level allele frequency of allele *A*. In an IGE, we collect two types of data for each individual: (1) genotypes (*G*_*i*_,*i *= 1,...,*n*), and (2) pairs of intensities of two alleles {(*S*_1*i*_,*S*_2*i*_),*i *= 1,...,*n*}. In a PAE, we collect a pair of intensities of two alleles (*S*_1_, *S*_2_) in a DNA pool with a pool size of *n*.

First, we introduce the estimating procedures for an individual-level allele frequency, which is a proportion of a specific allele in a genotype. An individual-level allele frequency can be estimated using an allele-counting approach based on genotypic data and an intensity-measuring approach based on intensity data from an IGE.

The allele-counting approach uses genotype data from an IGE. The number of allele *A *is counted and then used to calculate the proportion of allele *A *in a genotype. Under the assumptions of Hardy-Weinberg equilibrium and random sampling, the formulae of the estimator of allele frequency of allele *A *and its expectation and variance can be written as follows:(A1)

where *I*[*E*] is an indicator taking a value of 1 if event *E *holds; otherwise, the value is 0.

The intensity-measuring approach uses "adjusted" intensity data from an IGE, where intensities are "adjusted" by considering a coefficient of preferential amplification/hybridization (CPA). Based on intensity data from individuals who are heterozygous at the SNP, CPA can be estimated as follows [6,12,17]:

where  are the sample means of intensities of individuals and *n*_heter _is the number of individual heterozygous for the SNP. For each individual, the frequency of allele *A *is estimated by calculating the ratio of adjusted intensities pertaining to allele *A *relative to the total intensity of the two alleles. The estimator and its expectation and variance can be written as follows:(A2)

where a first-order Taylor expansion is used to calculate of the approximate expectation and variance,  is the coefficient of variation of CPA, and *σ*^2 ^is the experimental variation.

Second, we introduce procedures to estimate a population-level allele frequency, which is a proportion of a specific allele in a study population. The allele frequencies can be estimated based on data from an IGE or a PAE. In an IGE, a population-level allele frequency can be estimated by taking an average over individual-level allele frequencies from genotype data (**Equation (A1)**) or intensity data (**Equation (A2)**). The estimators and their expectations and variances can be written as follows:(A3)(A4)

In a PAE, relative intensities of allele *A *in a DNA pool, which is constructed by mixing genomic DNA from *n *samples, are calculated to estimate a population-level allele frequency. The estimator and its expectation and variance can be written as follows:(A5)

### Appendix B. Identification of chromosomal aberrations

A sliding-window approach, aided by individual-level allele frequency plots, is developed to identify chromosomal aberrations. All SNPs are arranged in order of physical position on a study chromosome. Let  and denote the genotype data and the individual-level allele frequency of the *m*th SNP of the *i*th SNP chip. For any SNP *m*, the genotype-specific reference mean and standard deviation of the individual-level allele frequency can be calculated based on a large number of reference samples as follows:

where *G*_*m *_is *AA*, *Aa *or *aa*. Then three genotype-specific reference confidence intervals can be written as follows:

. The first single-point index  is used to detect an allelic-imbalance (AI) SNP with allele frequency outside the allele frequency confidence intervals of the three genotypes. The second single-point index  is used to detect a loss-of-heterozygosity (LOH) or long-contiguous-stretch-of-homozygosity (LCSH) SNP with allele frequency outside the allele frequency confidence intervals of a heterozygous call. In other words, SNP *m *of individual *i *is identified as an AI SNP point if *A*_*i*,*m *_= 1 and identified as an LOH/LCSH SNP point if *L*_*i*,*m *_= 1. Two multilocus sliding-window chromosomal aberration detectors are developed as follows. The AI index can be written as(B1)

and the LOH/LCSH index as(B2)

where  denotes a study window with an anchor SNP *m *and  denotes the window size, i.e., the number of SNPs within the window. The indices are calculated for all normal controls and patients. We calculate a 95%-quantile of normal samples for index (*w*) and for index (*w*). The smoothed indices,  and , are calculated using a spline smoothing or local fit technique, such as a penalized regression [37]. We identify unusual genomic regions where the indices of a patient are higher than the 95%-quantile indices of normal controls in the human genome. The methods are useful for quickly scanning the human genome and accurately determining the starting and ending positions of chromosomal aberration regions for every patient.

A chromosomal aberration plot consisting of three components is used to detect special genomic patterns such as aneuploidy, LOH, and AI (Figures 2 and 3). The first component is an extended allele frequency plot where AI SNPs (= 1) are depicted by red points and non-AI SNPs (= 0) are depicted by blue points. The second component is an AI plot where index (*w*) of a patient and the 95% quantile of (*w*) of normal samples are displayed as light-red points and light-blue points, respectively, and smoothed versions are displayed as deep-red points and deep-blue points, respectively. The top panel shows a red bar that denotes an AI region if  is greater than the 95% quantile of  of normal samples; otherwise, a blue bar is shown. The third component is a LOH plot where index (*w*) of a patient and the 95% quantile of (*w*) of normal samples are displayed at light-red points and light-blue points, respectively, and smoothed versions are displayed as deep-red and deep-blue points, respectively. The top panel shows a red bar that denotes an LOH region if  is greater than the 95% quantile of  of normal samples; otherwise, a blue bar is shown.

### Appendix C. Allele frequency biplot

The biplot invented by Gabriel [38] was applied to explore microarray gene expression data [39,40] and LOH [41]. This paper first applies a biplot to visualize individual-level allele frequency data for hundreds of thousands of SNPs and large samples. Let **A **denote the individual-level allele frequency matrix with *R *rows (SNPs) and *C *columns (samples). Let **F **= **A **- (1/*C*)·**A**·**1**, where **1 **is a *C *× 1 column vector with cell values of 1. The matrix **F **is decomposed into a multiplication of an *R *× *K *SNP-effect matrix (row-effect matrix) **R **and a *C *× *K *sample-effect matrix (column-effect matrix) **C **using a singular value decomposition as outlined below. First, allele frequency matrix **F **is decomposed by a singular value decomposition as follows:(C1)

**P **is an *R *× *R *orthogonal matrix (i.e., **PP' **= **I**_*R*_), and the columns of **P **are eigenvectors of **FF'**; **Q **is an  orthogonal matrix (i.e., **QQ' **= **I**_*R *_and **QQ' **= **I**_*C*_) and the columns of **Q **are eigenvectors of **F'F**; **D **is an *R *× *R *diagonal matrix having *w *nonnegative elements as the square root of nonnegative eigenvalues of **FF' **and **F'F**, where . Second, allele frequency matrix **F **is further partitioned into row-effect and column-effect matrices as follows:(C2)

where **R **= **P **and **C **= **DQ **for a SNP-effect biplot, and **R **= **PD **and **C **= **Q **for a sample-effect biplot. Note that **FF' **= **RR' **for a SNP-effect biplot and **FF' **= **CC' **for a sample-effect biplot. For the convenience of visualization, a rank-2 approximate biplot is considered. Namely, the first two columns of **R **and **C **with the largest singular values are selected to approximate the allele frequency matrix **F**. A higher-rank approximate biplot is also plausible by visualizing more than two components pairwise.

### Appendix D. Estimation of the level of contamination by "normal" cells

Individual-level allele frequency can also be used to calculate the level of contaminating "normal" cells. By extending the previous method [42], we derive a general formula to estimate the proportion of contamination by normal cells for tumor cell samples using individual-level allele frequencies. Suppose that the study DNA is composed of 100(1-*p*)% tumor cells and 100*p*% contaminating "normal" cells. In normal cells without AI, the ratio of two alleles of a heterozygous SNP is 1 : 1; in cancer cells with AI, the ratio of two alleles is *a *: *b*, where *a *and *b *are the copies of the two alleles. Then the frequency of the first allele is *f*_1_=[*p*+*a*×(1-*p*)]/{[2-(*a*+*b*)]×*p*+(*a*+*b*)} and the frequency of the second allele is *f*_2_=[(1-*b*)×*p*+*b*]/{[2-(*a*+*b*)]×*p*+(*a*+*b*)}. Note that *p*, *a*, and *b *cannot be zero simultaneously. Using simple algebra, the normal cell contamination proportion is derived to be *p*=[*a*×(1- *f*_1_)-*b*×*f*_1_]/[(1- *b*)×*f*_1_)-(1-*a*)×*f*_2_] if (1-*a*)×*f*_2 _≠ (1-*b*)×*f*_1_. As an example of a hemizygous deletion of the first allele (i.e., *a *= 0, *b *= 1), then *f*_1_=*p*/(1+*p*), *f*_2 _= 1/(1+*p*) and *p *= *f*_1_/(1-*f*_1_). As an example of a single copy gain of the first allele (i.e., *a *= 2, *b *= 1), then *f*_1_=(2-*p*)/(3-*p*), *f*_2 _= 1/(3-*p*) and *p=*(2-3*f*_1_)/(1-*f*_1_).

## Supplementary Material

Additional file 1 **Figure S1.--Allele frequency of an individual (NA18996) from the JPT population based on the Affymetrix Human Mapping 100K Set**. This figure consists of 23 subfigures. Each subfigure presents an allele frequency plot of one chromosome. The vertical axis is the estimated allele frequency, and the horizontal axis is physical position (Mb). Each point denotes a SNP, and the gap in each subplot represents the centromeric gap. (A) Estimated allele frequency using an intensity-measuring approach. (B) Estimated allele frequency using an allele-counting approach. Click here for file

Additional file 2 **Figure S2--Unadjusted and adjusted individual-level allele frequency of a sample (NA18940) from the JPT population based on the Affymetrix Human Mapping 100K Set**. This figure consists of 23 subfigures. Each subfigure presents an allele frequency plot of one chromosome. The vertical axis is the estimated allele frequency, and the horizontal axis is physical position (Mb). Each point denotes a SNP, and the gap in each subplot represents the centromeric gap. (A) Unadjusted individual-level allele frequency estimates. (B) CPA-adjusted individual-level allele frequency estimates. Click here for file

Additional file 3 **Figure S3.--Unadjusted and adjusted population-level allele frequency of an artificial DNA pool with a size of 240 individuals based on the Affymetrix Human Mapping 100K Set**. The vertical axis is the estimated allele frequency, and the horizontal axis is the true allele frequency. (A) Unadjusted population-level allele frequency estimates. (B) CPA-adjusted population-level allele frequency estimates. Click here for file

Additional file 4 **Figure S4.--Genomic distributions of CPA in log_2 _scale and standard error of CPA for four sample sizes**. (A) This figure consists of 16 subfigures. The four diagonal subfigures are the histograms of log_2_(CPA) for sample sizes of 367, 180, 90 and 45. The off-diagonal subfigures are scatter plots of log_2_(CPA) for pairs of sample sizes, where each blue point denotes a log_2_(CPA) value of a SNP. A quadratic regression curve is fitted. The mean regression curve is plotted in red, and the corresponding 95% confidence interval is plotted in green. (B) The figure contains ratios of CPA standard errors of 180 versus 367 samples (green points), 90 versus 367 samples (red points) and 45 versus 367 samples (blue points). The red reference line denotes the ratio of 1, i.e., equal to the CPA standard error of 367 samples. Click here for file

Additional file 5 **Figure S5.--Genomic distributions of CPA in log_2 _scale for different data acquisition times (four genotyping periods) and experimental sites (two laboratories)**. (A) This figure consists of 16 subfigures. The four diagonal subfigures are the histograms of log_2_(CPA) for the genotyping done on the time 2005/05/04, 2006/01/09, 2006/03/17 and 2006/06/29. The off-diagonal subfigures are scatter plots of log_2_(CPA) for pairs of genotyping periods, where each blue point denotes a log_2_(CPA) value of a SNP. (B) This figure consists of 23 subfigures. Each subfigure shows a scatter plot of CPAs in log_2 _scale of one chromosome based on 90 Asian samples in the HapMap project (vertical axis) and 95 Taiwanese samples (horizontal axis). A quadratic mean regression curve (red) and the corresponding 95% confidence intervals (green) are calculated. Click here for file

Additional file 6 **Figure S6.--Genomic distributions of CPA in log_2 _scale based on the Affymetrix Human Mapping 100K and 500K Sets**. This figure consists of 23 subfigures. Each subfigure shows a scatter plot or histogram of CPAs in log_2 _scale of one chromosome. (A) Scatter plots of CPAs in log_2 _scale for the Affymetrix Human Mapping 100K Set based on 457 Asian samples. (B) Histograms of CPAs in log_2 _scale for the Affymetrix Human Mapping 100K Set based on 457 Asian samples. (C) Scatter plots of CPAs in log_2 _scale for the Affymetrix Human Mapping 500K Set based on 538 Asian samples. (D) Histograms of CPAs in log_2 _scale for the Affymetrix Human Mapping 500K Set based on 538 Asian samples. Click here for file

Additional file 7 **Figure S7.--Lognormal distribution of CPA based on Taiwanese samples**. CPAs are fitted using log-normal distributions by chromosome. The green curve is a fitted curve, and the purple curve is a theoretical lognormal curve. (A) Taiwanese samples (367 in total) that were genotyped with the Affymetrix Human Mapping 100K Set. (B) Taiwanese samples (448 in total) that were genotyped with the Affymetrix Human Mapping 500K Set. Click here for file

Additional file 8 **Figure S8.--Genomic distributions of CPA in log_2 _scale for different ethnic groups and a tree diagram**. (A) This figure consists of 16 subfigures. The six diagonal subfigures are the histograms of log_2_(CPA) for 45 CHB, 45 JPT, 60 CEU founders, 60 YRI founders, 90 Asians (45 CHB and 45 JPT), and 210 combined samples. The off-diagonal subfigures are scatter plots of log_2_(CPA) for pairs of groups, where each blue point denotes a log_2_(CPA) value of a SNP. A quadratic mean regression curve (red) and the corresponding 95% confidence intervals (green) are provided. (B) The studied populations are clustered according to between-population proximity (CPA correlation) via an average-linkage clustering analysis. Click here for file

Additional file 9 **Figure S9.--Genomic distributions of CPA in log_2 _scale for hypertensive case group and normotensive control group**. This figure consists of 23 subfigures. Each subfigure shows a scatter plot of CPAs in log_2 _scale of one chromosome based on data from 175 hypertension patient samples (vertical axis) and 180 normal control samples (horizontal axis). A quadratic mean regression curve (red) and the corresponding 95% confidence intervals (green) are shown. Click here for file

Additional file 10 **Figure S10.--Allele frequency biplots of sex chromosomes of HapMap samples based on the Affymetrix Human Mapping 500K Set**. (A) Allele frequency biplots of sex chromosomes for YRI populations in HapMap (30 fathers and 30 mothers in YRI). (B) Allele frequency biplots of sex chromosomes for CEU populations in HapMap (30 fathers and 30 mothers in CEU). Click here for file

Additional file 11 **Figure S11.--Allele frequency of a normal sample based on the Illumina HumanHap550-Duo BeadChip**. This figure consists of 23 subfigures. Each subfigure presents an allele frequency plot of one chromosome. The vertical axis is the estimated allele frequency, and the horizontal axis is physical position (Mb). Each point denotes a SNP, and the gap in each subplot represents the centromeric gap. The allele frequencies were estimated using an intensity-measuring approach. Click here for file
