# Supplementary material for: A new analysis tool for individual-level allele frequency for genomic studies
Source: BMC Genomics. 2010 Jul 5;11:415. doi: 10.1186/1471-2164-11-415 (PMC2996943; doi:10.1186/1471-2164-11-415)

**Figure S1.—Allele frequency of an individual (NA18996) from the JPT population based on the Affymetrix Human Mapping 100K Set.** This figure consists of 23 subfigures. Each subfigure presents an allele frequency plot of one chromosome. The vertical axis is the estimated allele frequency, and the horizontal axis is physical position (Mb). Each point denotes a SNP, and the gap in each subplot represents the centromeric gap. (A) Estimated allele frequency using an intensity-measuring approach. (B) Estimated allele frequency using an allele-counting approach.

**(A)**


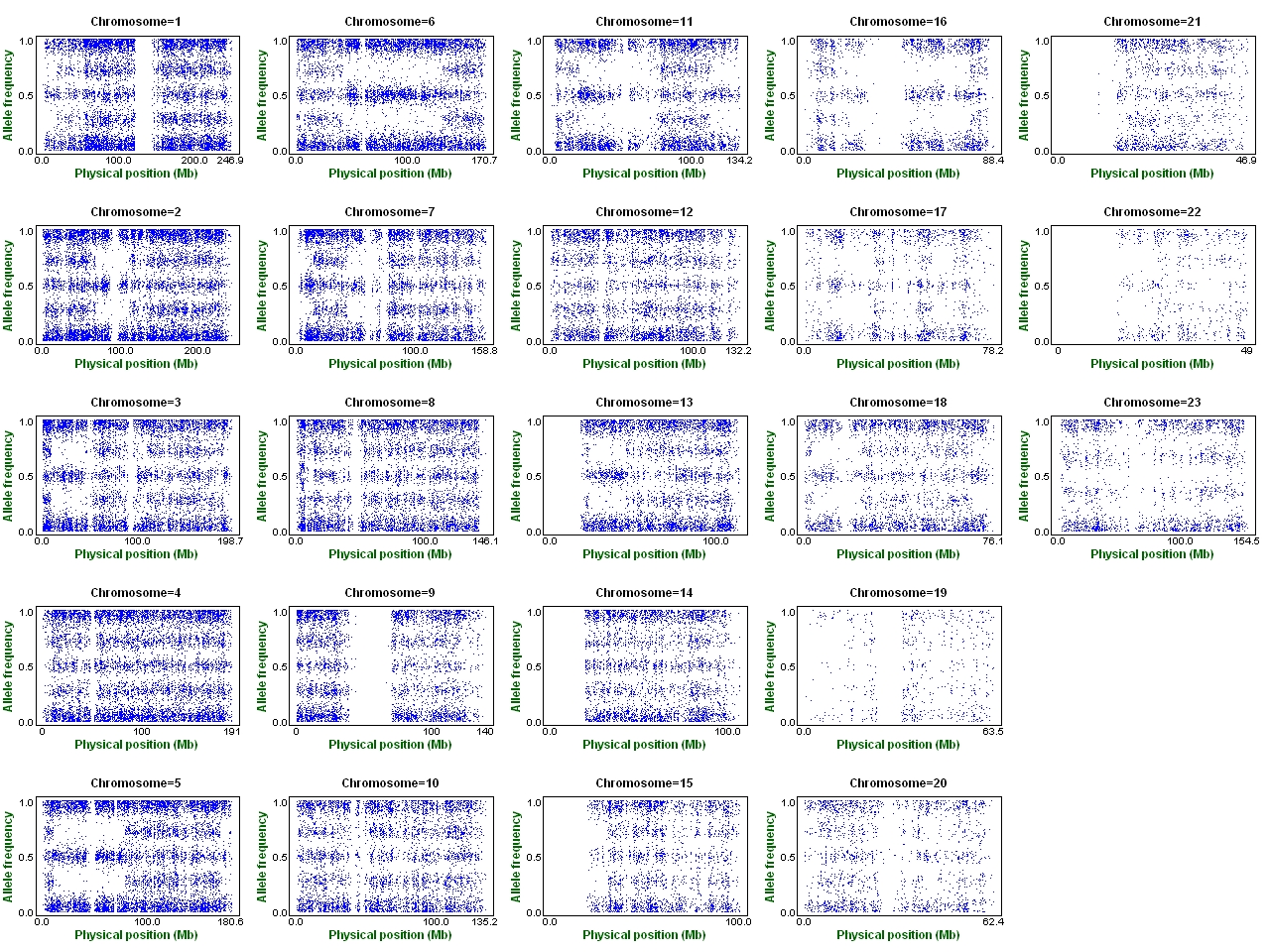


**(B)**


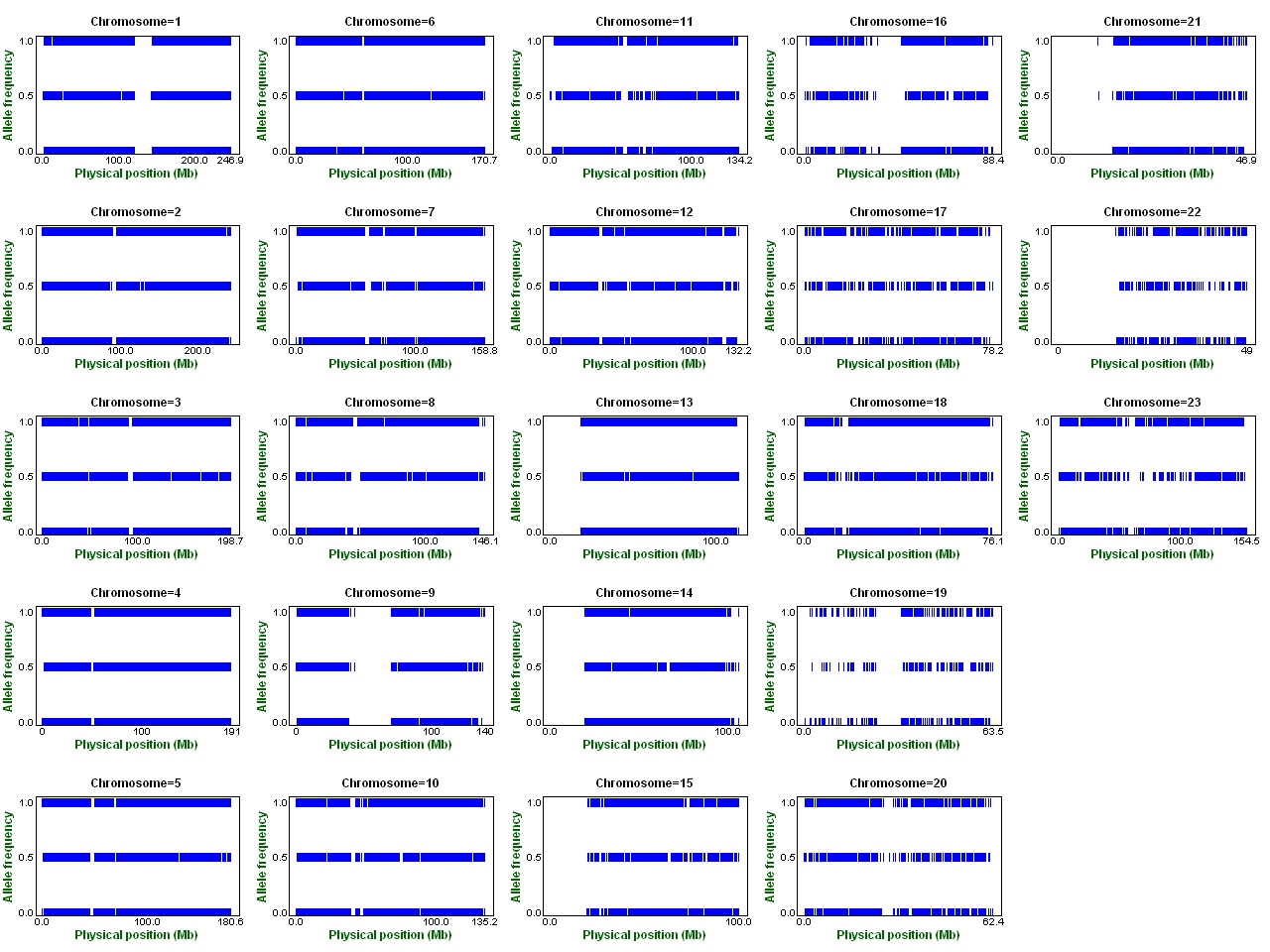

Supplement: Additional file 1 — Figure S1.--Allele frequency of an individual (NA18996) from the JPT population based on the Affymetrix Human Mapping 100K Set. This figure consists of 23 subfigures. Each subfigure presents an allele frequency plot of one chromosome. The vertical axis is the estimated allele frequency, and the horizontal axis is physical position (Mb). Each point denotes a SNP, and the gap in each subplot represents the centromeric gap. (A) Estimated allele frequency using an intensity-measuring approach. (B) Estimated allele frequency using an allele-counting approach. [file 1471-2164-11-415-S1.DOC]
