# Supplementary material for: A new analysis tool for individual-level allele frequency for genomic studies
Source: BMC Genomics. 2010 Jul 5;11:415. doi: 10.1186/1471-2164-11-415 (PMC2996943; doi:10.1186/1471-2164-11-415)
Supplement: Additional file 2 — Figure S2--Unadjusted and adjusted individual-level allele frequency of a sample (NA18940) from the JPT population based on the Affymetrix Human Mapping 100K Set. This figure consists of 23 subfigures. Each subfigure presents an allele frequency plot of one chromosome. The vertical axis is the estimated allele frequency, and the horizontal axis is physical position (Mb). Each point denotes a SNP, and the gap in each subplot represents the centromeric gap. (A) Unadjusted individual-level allele frequency estimates. (B) CPA-adjusted individual-level allele frequency estimates. [file 1471-2164-11-415-S2.DOC]

**Figure S2.**—**Unadjusted and adjusted individual-level allele frequency of a sample (NA18940) from the JPT population based on the Affymetrix Human Mapping 100K Set.** This figure consists of 23 subfigures. Each subfigure presents an allele frequency plot of one chromosome. The vertical axis is the estimated allele frequency, and the horizontal axis is physical position (Mb). Each point denotes a SNP, and the gap in each subplot represents the centromeric gap. (A) Unadjusted individual-level allele frequency estimates. (B) CPA-adjusted individual-level allele frequency estimates.

**(A)**

**
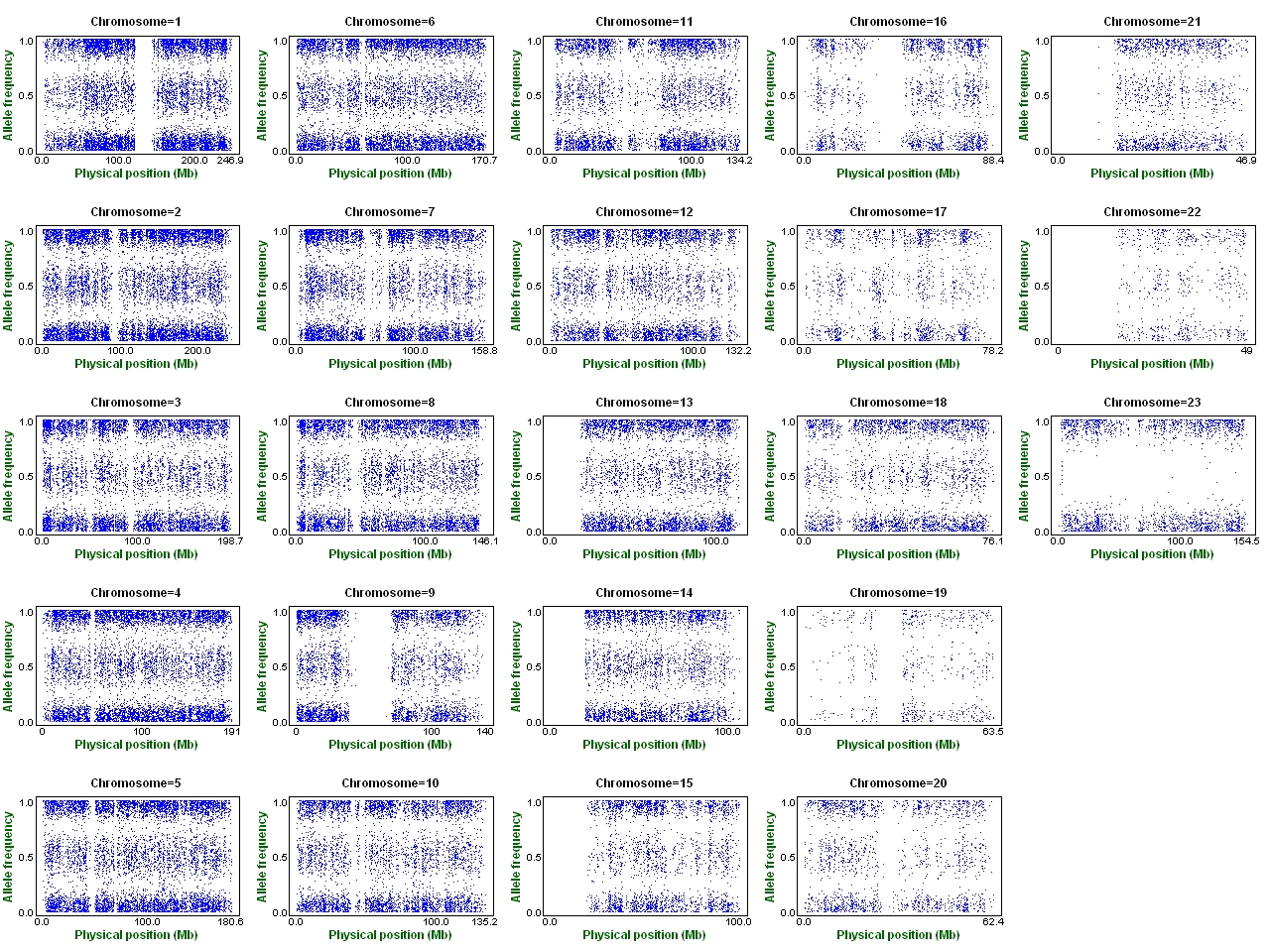
**

**(B)**

**
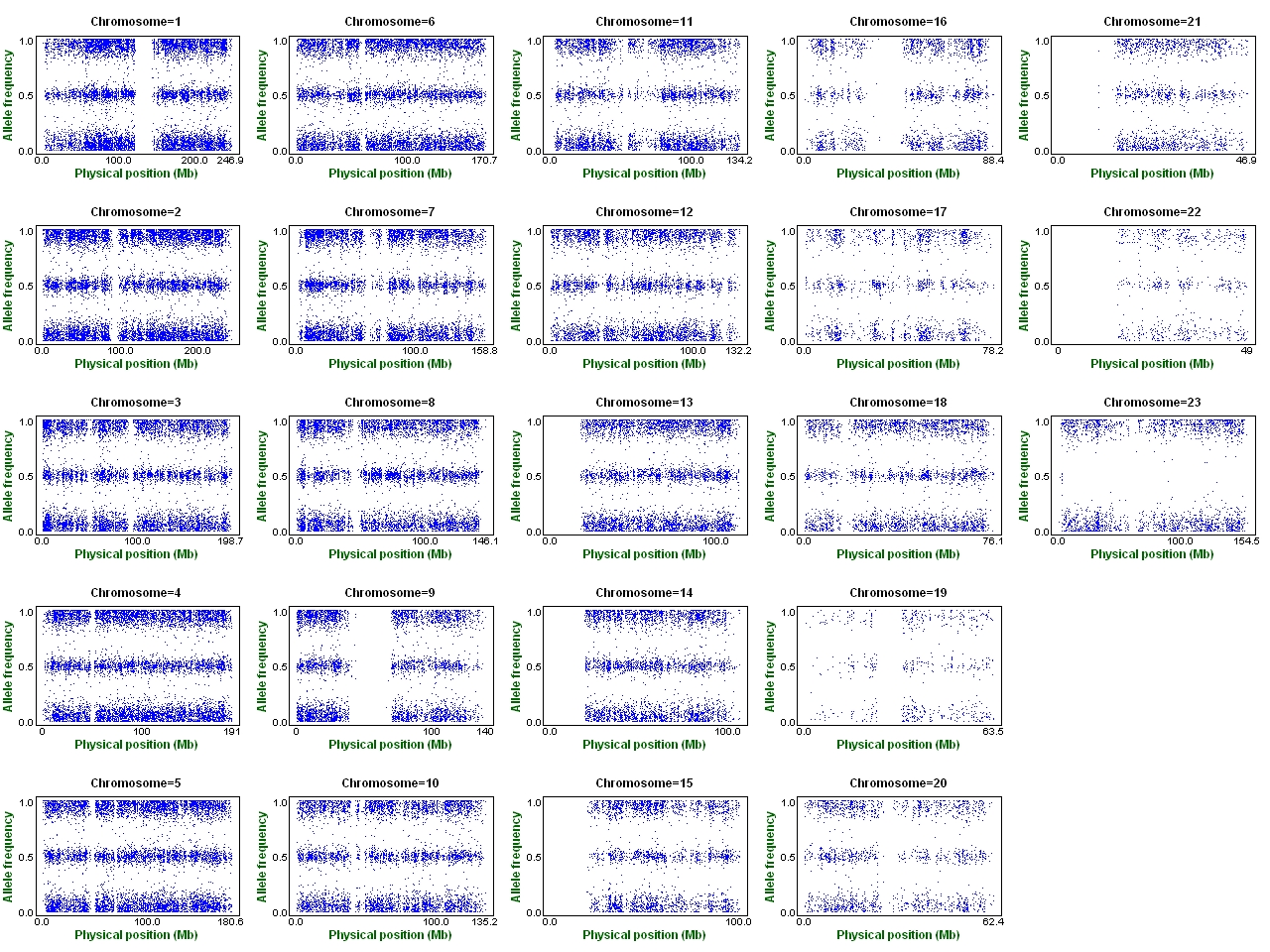
**
