# Supplementary material for: A new analysis tool for individual-level allele frequency for genomic studies
Source: BMC Genomics. 2010 Jul 5;11:415. doi: 10.1186/1471-2164-11-415 (PMC2996943; doi:10.1186/1471-2164-11-415)

**Figure S3.**—**Unadjusted and adjusted population-level allele frequency of an artificial DNA pool with a size of 240 individuals based on the Affymetrix Human Mapping 100K Set.** The vertical axis is the estimated allele frequency, and the horizontal axis is the true allele frequency. (A) Unadjusted population-level allele frequency estimates. (B) CPA-adjusted population-level allele frequency estimates.

**(A)**


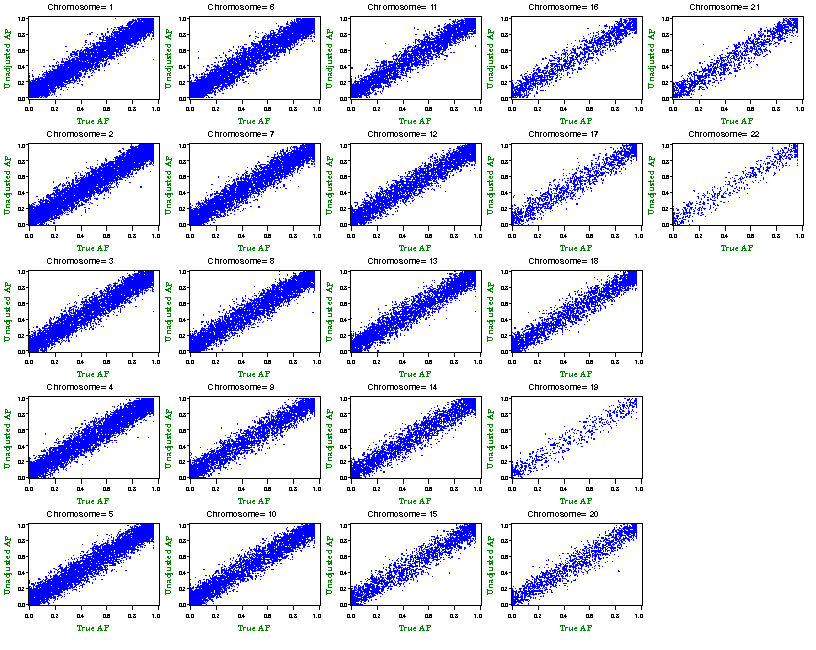


**(B)**


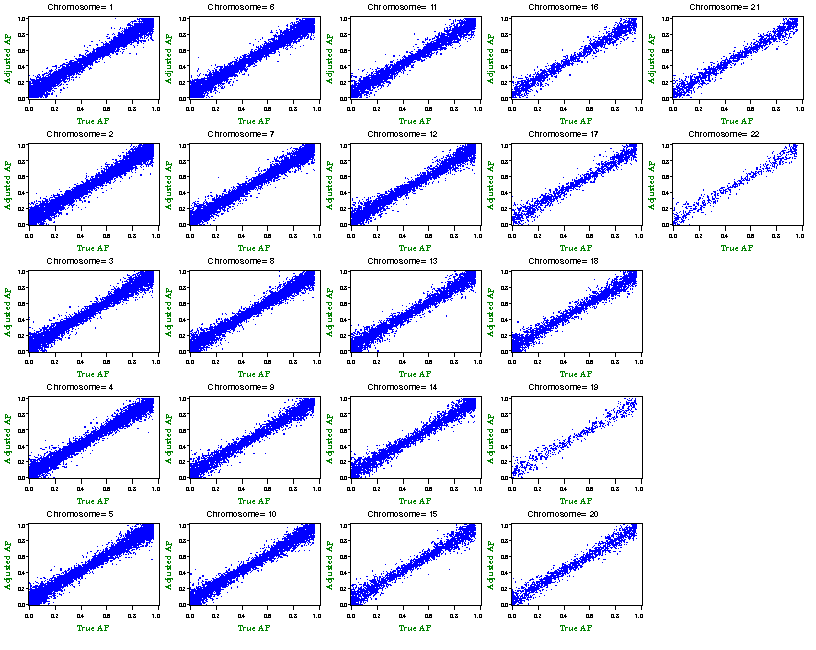

Supplement: Additional file 3 — Figure S3.--Unadjusted and adjusted population-level allele frequency of an artificial DNA pool with a size of 240 individuals based on the Affymetrix Human Mapping 100K Set. The vertical axis is the estimated allele frequency, and the horizontal axis is the true allele frequency. (A) Unadjusted population-level allele frequency estimates. (B) CPA-adjusted population-level allele frequency estimates. [file 1471-2164-11-415-S3.DOC]
