# Supplementary material for: A new analysis tool for individual-level allele frequency for genomic studies
Source: BMC Genomics. 2010 Jul 5;11:415. doi: 10.1186/1471-2164-11-415 (PMC2996943; doi:10.1186/1471-2164-11-415)
Supplement: Additional file 4 — Figure S4.--Genomic distributions of CPA in log2 scale and standard error of CPA for four sample sizes. (A) This figure consists of 16 subfigures. The four diagonal subfigures are the histograms of log2(CPA) for sample sizes of 367, 180, 90 and 45. The off-diagonal subfigures are scatter plots of log2(CPA) for pairs of sample sizes, where each blue point denotes a log2(CPA) value of a SNP. A quadratic regression curve is fitted. The mean regression curve is plotted in red, and the corresponding 95% confidence interval is plotted in green. (B) The figure contains ratios of CPA standard errors of 180 versus 367 samples (green points), 90 versus 367 samples (red points) and 45 versus 367 samples (blue points). The red reference line denotes the ratio of 1, i.e., equal to the CPA standard error of 367 samples. [file 1471-2164-11-415-S4.DOC]

**Figure S4.**—**Genomic distributions of CPA in log_2_ scale and standard error of CPA for four sample sizes.** (A) This figure consists of 16 subfigures. The four diagonal subfigures are the histograms of log_2_(CPA) for sample sizes of 367, 180, 90 and 45. The off-diagonal subfigures are scatter plots of log_2_(CPA) for pairs of sample sizes, where each blue point denotes a log_2_(CPA) value of a SNP. A quadratic regression curve is fitted. The mean regression curve is plotted in red, and the corresponding 95% confidence interval is plotted in green. (B) The figure contains ratios of CPA standard errors of 180 versus 367 samples (green points), 90 versus 367 samples (red points) and 45 versus 367 samples (blue points). The red reference line denotes the ratio of 1, i.e., equal to the CPA standard error of 367 samples.

**(A)**

**
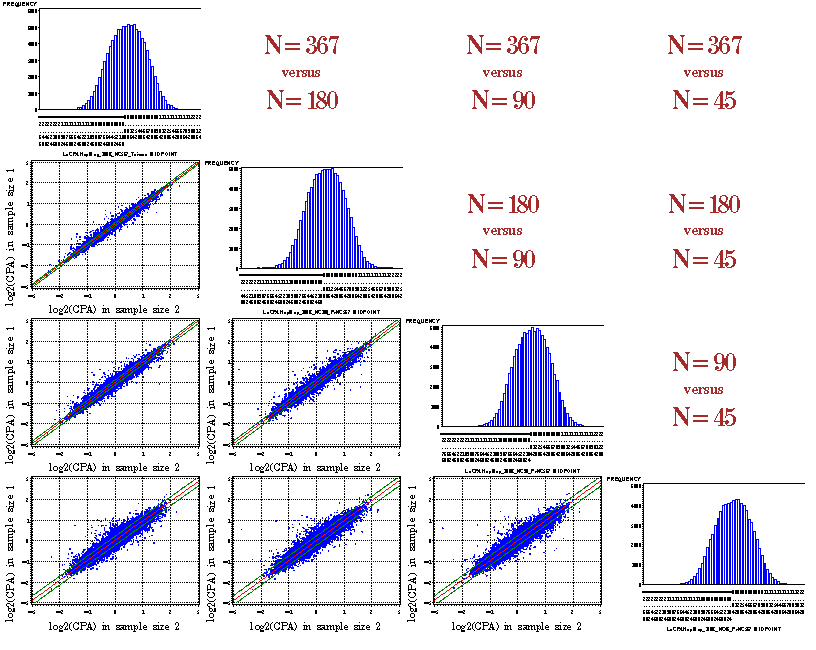
**

**(B)**

**
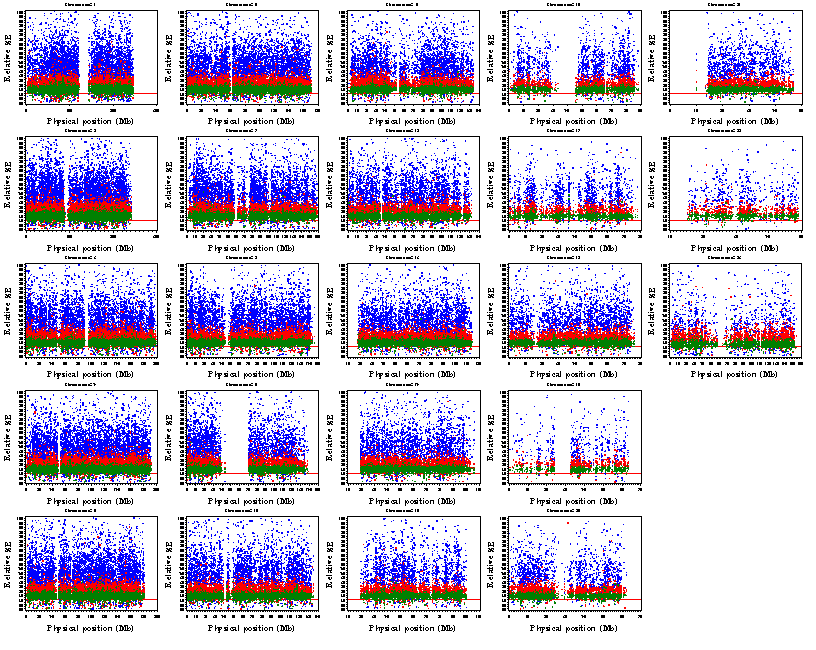
**
