# Supplementary material for: A new analysis tool for individual-level allele frequency for genomic studies
Source: BMC Genomics. 2010 Jul 5;11:415. doi: 10.1186/1471-2164-11-415 (PMC2996943; doi:10.1186/1471-2164-11-415)

**Figure S5.**—**Genomic distributions of CPA in log_2_ scale for different data acquisition times (four genotyping periods) and experimental sites (two laboratories).** (A) This figure consists of 16 subfigures. The four diagonal subfigures are the histograms of log_2_(CPA) for the genotyping done on the time 2005/05/04, 2006/01/09, 2006/03/17 and 2006/06/29. The off-diagonal subfigures are scatter plots of log_2_(CPA) for pairs of genotyping periods, where each blue point denotes a log_2_(CPA) value of a SNP. (B) This figure consists of 23 subfigures. Each subfigure shows a scatter plot of CPAs in log_2_ scale of one chromosome based on 90 Asian samples in the HapMap project (vertical axis) and 95 Taiwanese samples (horizontal axis). A quadratic mean regression curve (red) and the corresponding 95% confidence intervals (green) are calculated.

**(A)**

**
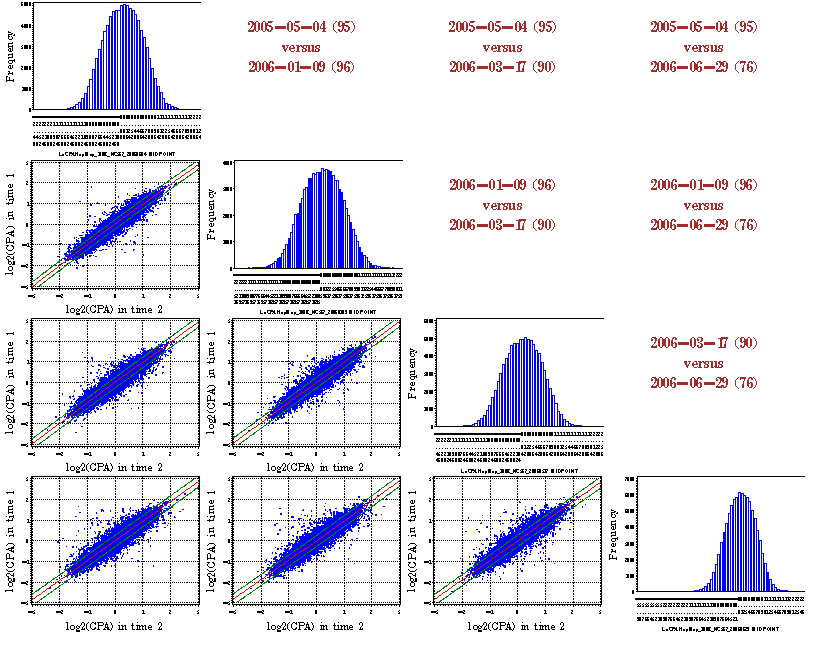
**

**(B)**


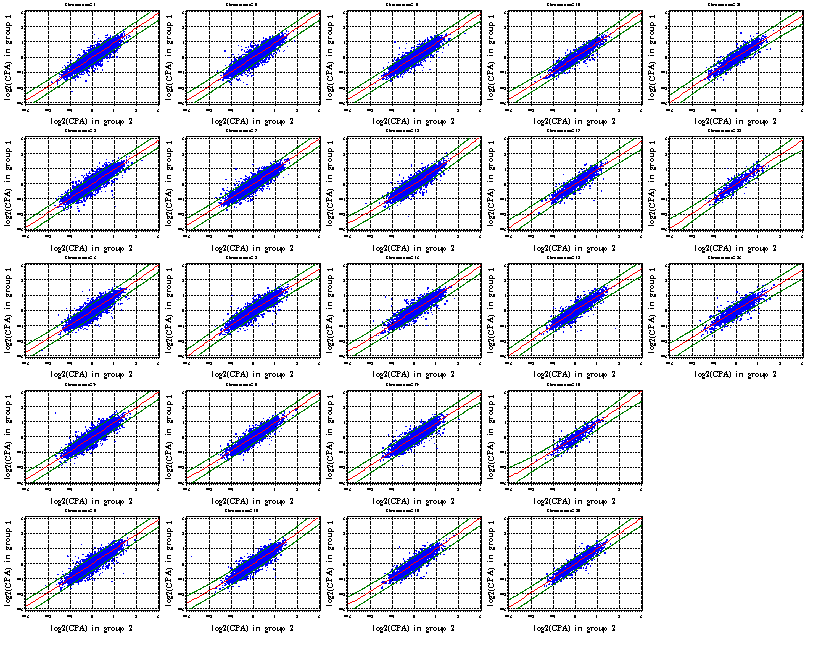

Supplement: Additional file 5 — Figure S5.--Genomic distributions of CPA in log2 scale for different data acquisition times (four genotyping periods) and experimental sites (two laboratories). (A) This figure consists of 16 subfigures. The four diagonal subfigures are the histograms of log2(CPA) for the genotyping done on the time 2005/05/04, 2006/01/09, 2006/03/17 and 2006/06/29. The off-diagonal subfigures are scatter plots of log2(CPA) for pairs of genotyping periods, where each blue point denotes a log2(CPA) value of a SNP. (B) This figure consists of 23 subfigures. Each subfigure shows a scatter plot of CPAs in log2 scale of one chromosome based on 90 Asian samples in the HapMap project (vertical axis) and 95 Taiwanese samples (horizontal axis). A quadratic mean regression curve (red) and the corresponding 95% confidence intervals (green) are calculated. [file 1471-2164-11-415-S5.DOC]
