# Supplementary material for: A new analysis tool for individual-level allele frequency for genomic studies
Source: BMC Genomics. 2010 Jul 5;11:415. doi: 10.1186/1471-2164-11-415 (PMC2996943; doi:10.1186/1471-2164-11-415)
Supplement: Additional file 6 — Figure S6.--Genomic distributions of CPA in log2 scale based on the Affymetrix Human Mapping 100K and 500K Sets. This figure consists of 23 subfigures. Each subfigure shows a scatter plot or histogram of CPAs in log2 scale of one chromosome. (A) Scatter plots of CPAs in log2 scale for the Affymetrix Human Mapping 100K Set based on 457 Asian samples. (B) Histograms of CPAs in log2 scale for the Affymetrix Human Mapping 100K Set based on 457 Asian samples. (C) Scatter plots of CPAs in log2 scale for the Affymetrix Human Mapping 500K Set based on 538 Asian samples. (D) Histograms of CPAs in log2 scale for the Affymetrix Human Mapping 500K Set based on 538 Asian samples. [file 1471-2164-11-415-S6.DOC]

**Figure S6.**—**Genomic distributions of CPA in log_2_ scale based on the Affymetrix Human Mapping 100K and 500K Sets.** This figure consists of 23 subfigures. Each subfigure shows a scatter plot or histogram of CPAs in log_2_ scale of one chromosome. (A) Scatter plots of CPAs in log_2_ scale for the Affymetrix Human Mapping 100K Set based on 457 Asian samples. (B) Histograms of CPAs in log_2_ scale for the Affymetrix Human Mapping 100K Set based on 457 Asian samples. (C) Scatter plots of CPAs in log_2_ scale for the Affymetrix Human Mapping 500K Set based on 538 Asian samples. (D) Histograms of CPAs in log_2_ scale for the Affymetrix Human Mapping 500K Set based on 538 Asian samples.

**(A)**

**
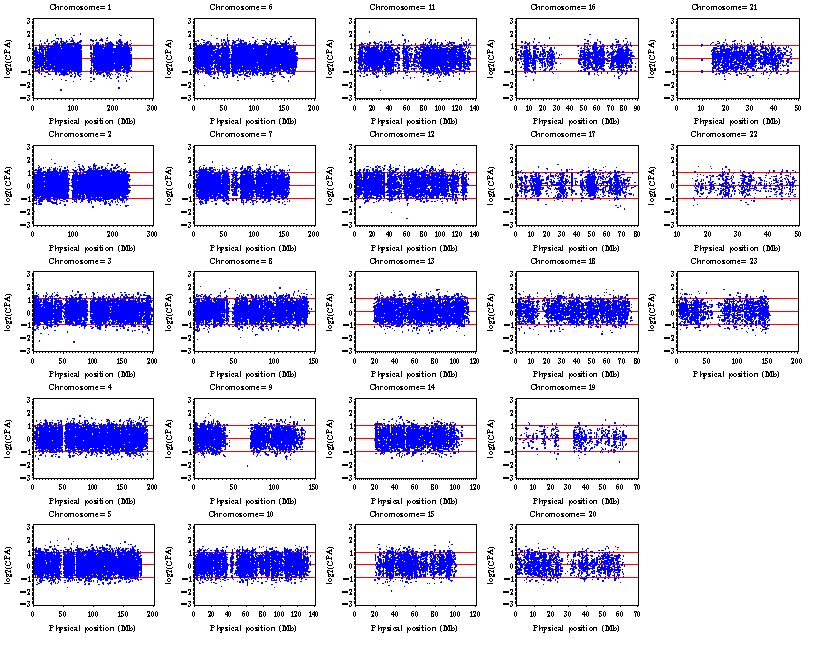
**

**(B)
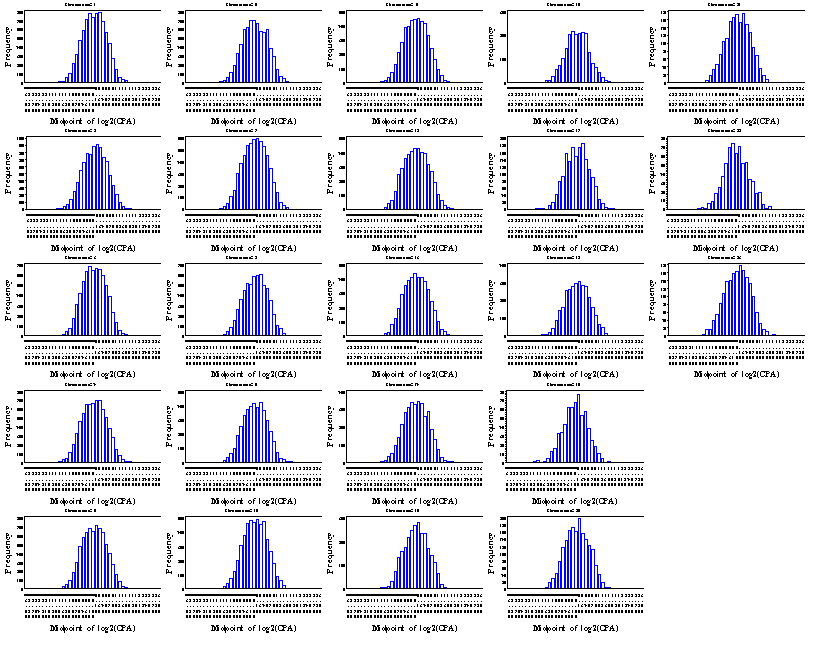
**

**(C)**

**
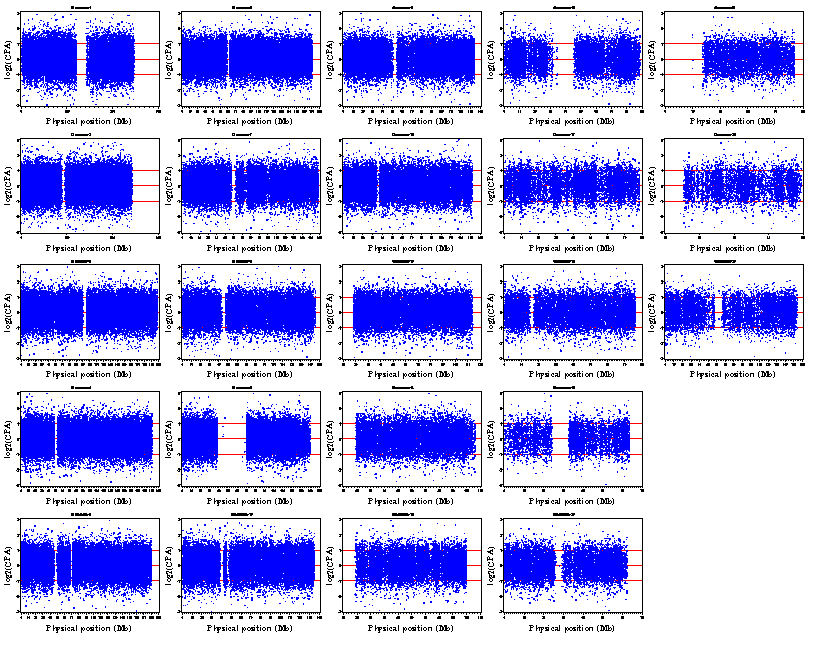
**

**(D)**

**
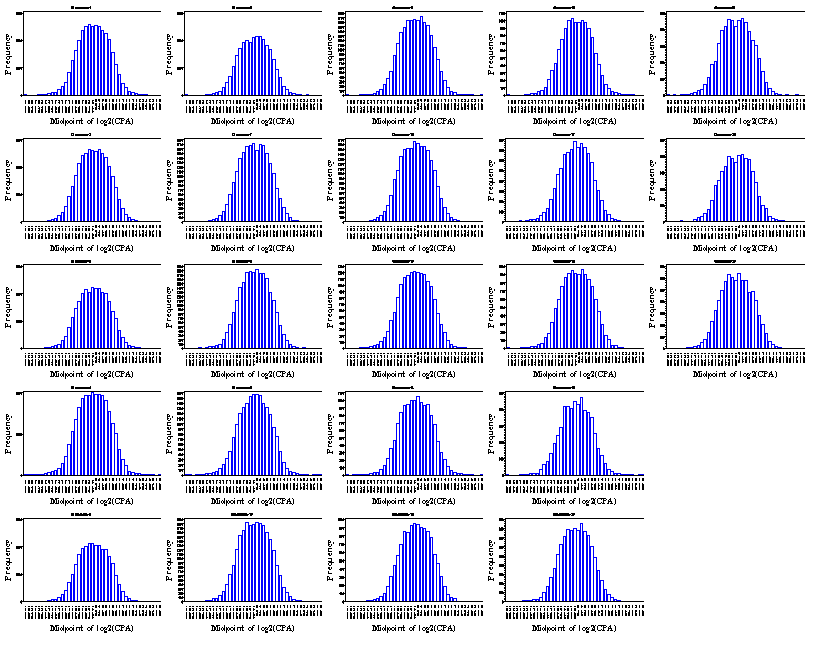
**
