# Supplementary material for: A new analysis tool for individual-level allele frequency for genomic studies
Source: BMC Genomics. 2010 Jul 5;11:415. doi: 10.1186/1471-2164-11-415 (PMC2996943; doi:10.1186/1471-2164-11-415)
Supplement: Additional file 7 — Figure S7.--Lognormal distribution of CPA based on Taiwanese samples. CPAs are fitted using log-normal distributions by chromosome. The green curve is a fitted curve, and the purple curve is a theoretical lognormal curve. (A) Taiwanese samples (367 in total) that were genotyped with the Affymetrix Human Mapping 100K Set. (B) Taiwanese samples (448 in total) that were genotyped with the Affymetrix Human Mapping 500K Set. [file 1471-2164-11-415-S7.DOC]

**Figure S7.**—**Lognormal distribution of CPA based on Taiwanese samples.** CPAs are fitted using log-normal distributions by chromosome. The green curve is a fitted curve, and the purple curve is a theoretical lognormal curve. (A) Taiwanese samples (367 in total) that were genotyped with the Affymetrix Human Mapping 100K Set. (B) Taiwanese samples (448 in total) that were genotyped with the Affymetrix Human Mapping 500K Set.

**(A)**

**
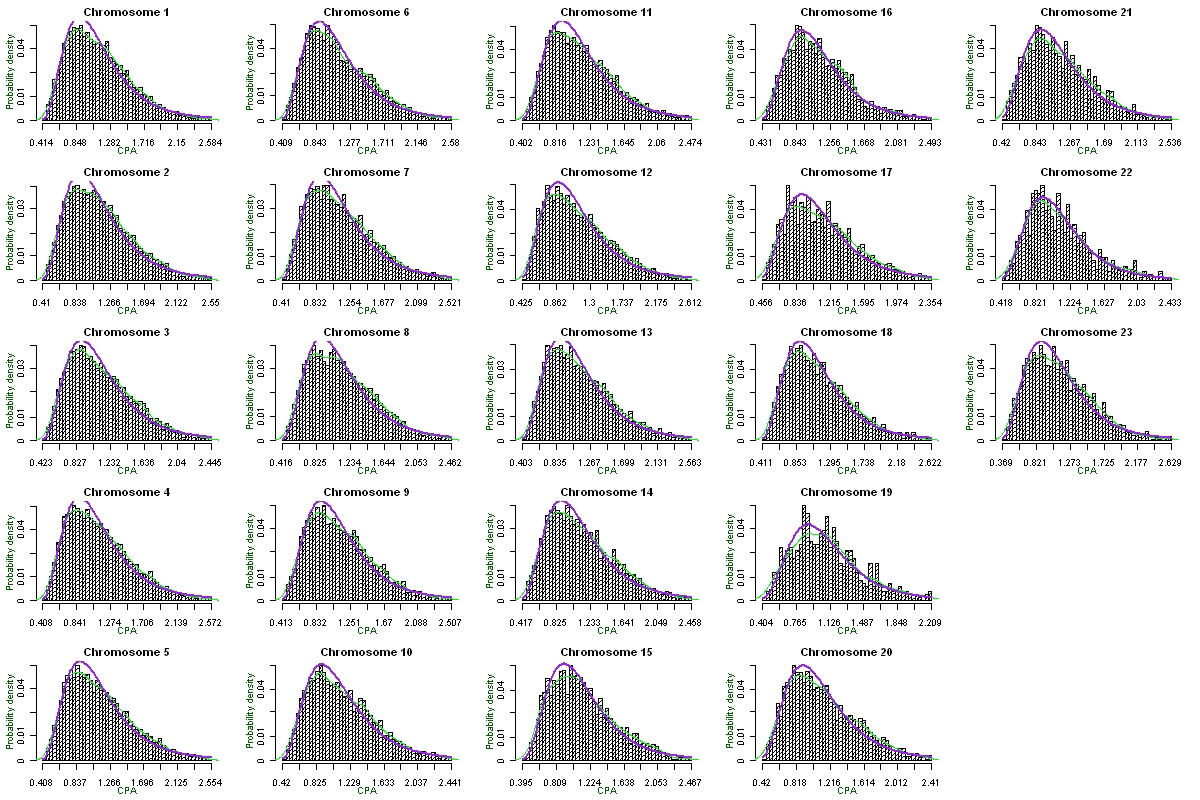
**

**(B)**

**
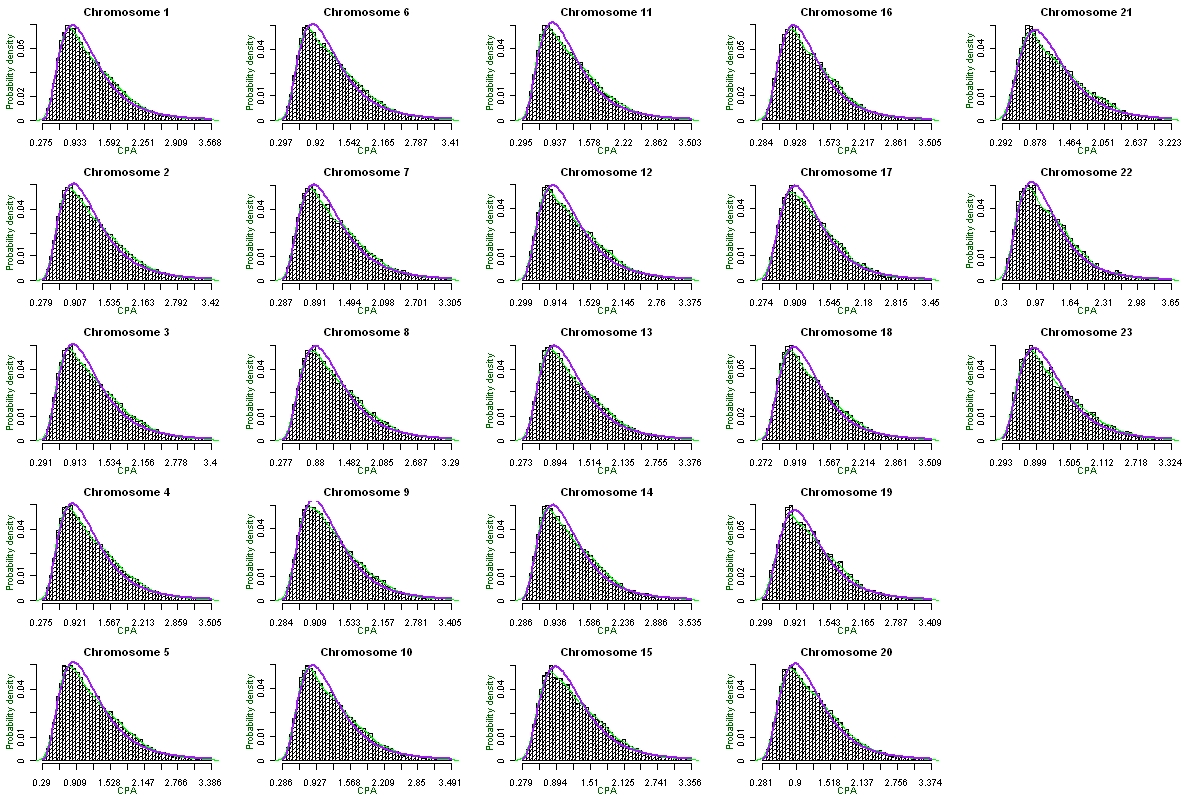
**
