# Supplementary material for: A new analysis tool for individual-level allele frequency for genomic studies
Source: BMC Genomics. 2010 Jul 5;11:415. doi: 10.1186/1471-2164-11-415 (PMC2996943; doi:10.1186/1471-2164-11-415)
Supplement: Additional file 8 — Figure S8.--Genomic distributions of CPA in log2 scale for different ethnic groups and a tree diagram. (A) This figure consists of 16 subfigures. The six diagonal subfigures are the histograms of log2(CPA) for 45 CHB, 45 JPT, 60 CEU founders, 60 YRI founders, 90 Asians (45 CHB and 45 JPT), and 210 combined samples. The off-diagonal subfigures are scatter plots of log2(CPA) for pairs of groups, where each blue point denotes a log2(CPA) value of a SNP. A quadratic mean regression curve (red) and the corresponding 95% confidence intervals (green) are provided. (B) The studied populations are clustered according to between-population proximity (CPA correlation) via an average-linkage clustering analysis. [file 1471-2164-11-415-S8.DOC]

**Figure S8.**—**Genomic distributions of CPA in log_2_ scale for different ethnic groups and a tree diagram.** (A) This figure consists of 16 subfigures. The six diagonal subfigures are the histograms of log_2_(CPA) for 45 CHB, 45 JPT, 60 CEU founders, 60 YRI founders, 90 Asians (45 CHB and 45 JPT), and 210 combined samples. The off-diagonal subfigures are scatter plots of log_2_(CPA) for pairs of groups, where each blue point denotes a log_2_(CPA) value of a SNP. A quadratic mean regression curve (red) and the corresponding 95% confidence intervals (green) are provided. (B) The studied populations are clustered according to between-population proximity (CPA correlation) via an average-linkage clustering analysis.

**(A)**

**
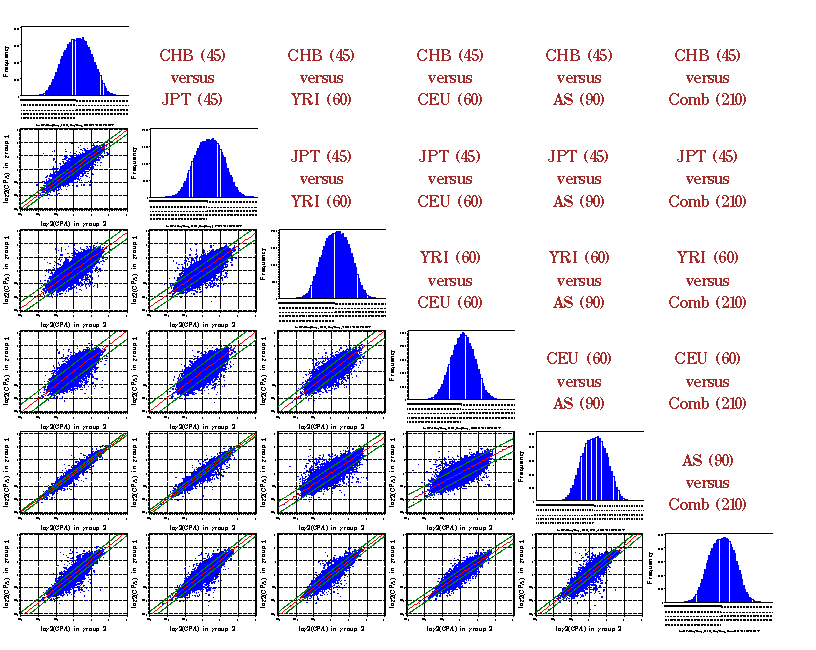
**

**(B)**

**
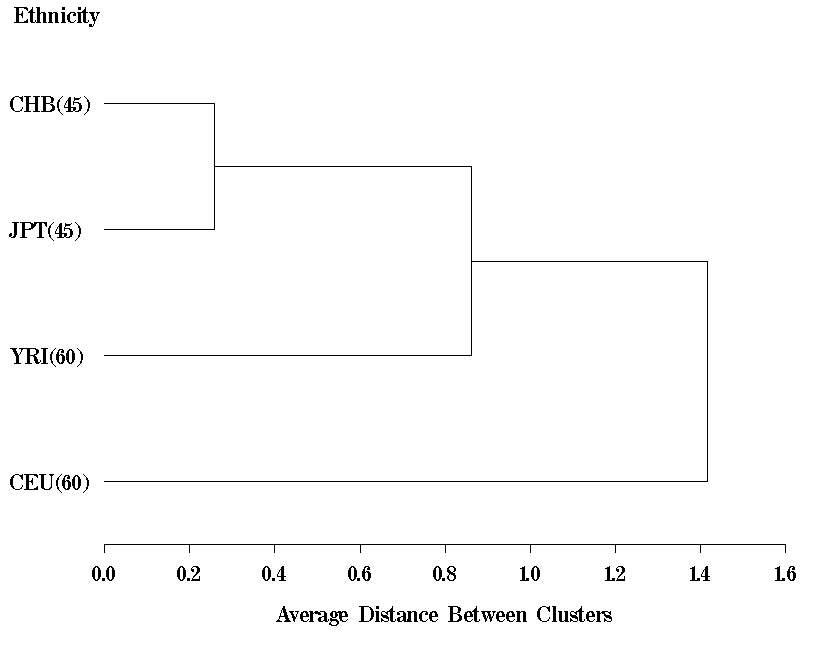
**
