# Supplementary material for: A new analysis tool for individual-level allele frequency for genomic studies
Source: BMC Genomics. 2010 Jul 5;11:415. doi: 10.1186/1471-2164-11-415 (PMC2996943; doi:10.1186/1471-2164-11-415)

**Figure S9.**—**Genomic distributions of CPA in log_2_ scale for hypertensive case group and normotensive control group.** This figure consists of 23 subfigures. Each subfigure shows a scatter plot of CPAs in log_2_ scale of one chromosome based on data from 175 hypertension patient samples (vertical axis) and 180 normal control samples (horizontal axis). A quadratic mean regression curve (red) and the corresponding 95% confidence intervals (green) are shown.


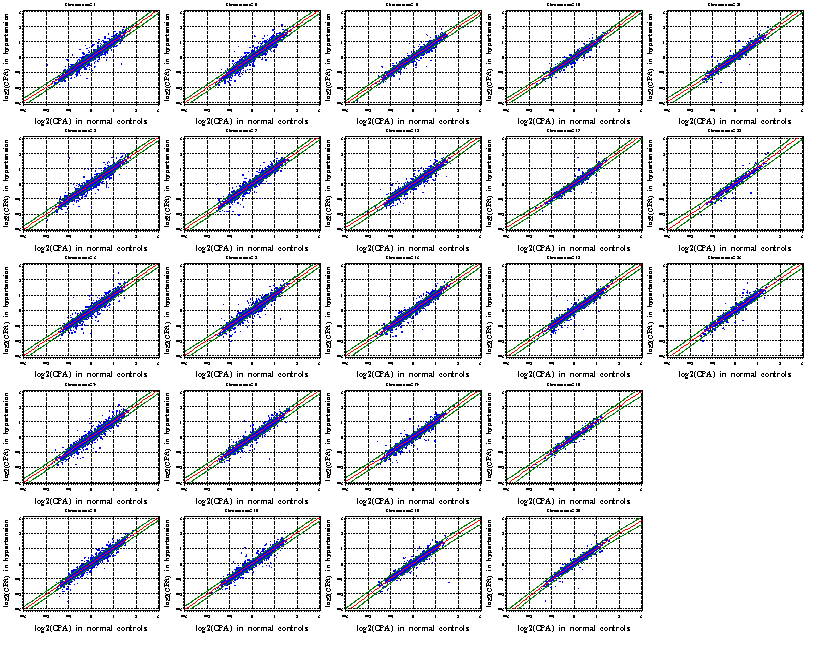

Supplement: Additional file 9 — Figure S9.--Genomic distributions of CPA in log2 scale for hypertensive case group and normotensive control group. This figure consists of 23 subfigures. Each subfigure shows a scatter plot of CPAs in log2 scale of one chromosome based on data from 175 hypertension patient samples (vertical axis) and 180 normal control samples (horizontal axis). A quadratic mean regression curve (red) and the corresponding 95% confidence intervals (green) are shown. [file 1471-2164-11-415-S9.DOC]
