# Supplementary material for: A new analysis tool for individual-level allele frequency for genomic studies
Source: BMC Genomics. 2010 Jul 5;11:415. doi: 10.1186/1471-2164-11-415 (PMC2996943; doi:10.1186/1471-2164-11-415)
Supplement: Additional file 10 — Figure S10.--Allele frequency biplots of sex chromosomes of HapMap samples based on the Affymetrix Human Mapping 500K Set. (A) Allele frequency biplots of sex chromosomes for YRI populations in HapMap (30 fathers and 30 mothers in YRI). (B) Allele frequency biplots of sex chromosomes for CEU populations in HapMap (30 fathers and 30 mothers in CEU). [file 1471-2164-11-415-S10.DOC]

**Figure S10.**—**Allele frequency biplots of sex chromosomes of HapMap samples based on the Affymetrix Human Mapping 500K Set.** (A) Allele frequency biplots of sex chromosomes for YRI populations in HapMap (30 fathers and 30 mothers in YRI). (B) Allele frequency biplots of sex chromosomes for CEU populations in HapMap (30 fathers and 30 mothers in CEU).

**(A)**

**
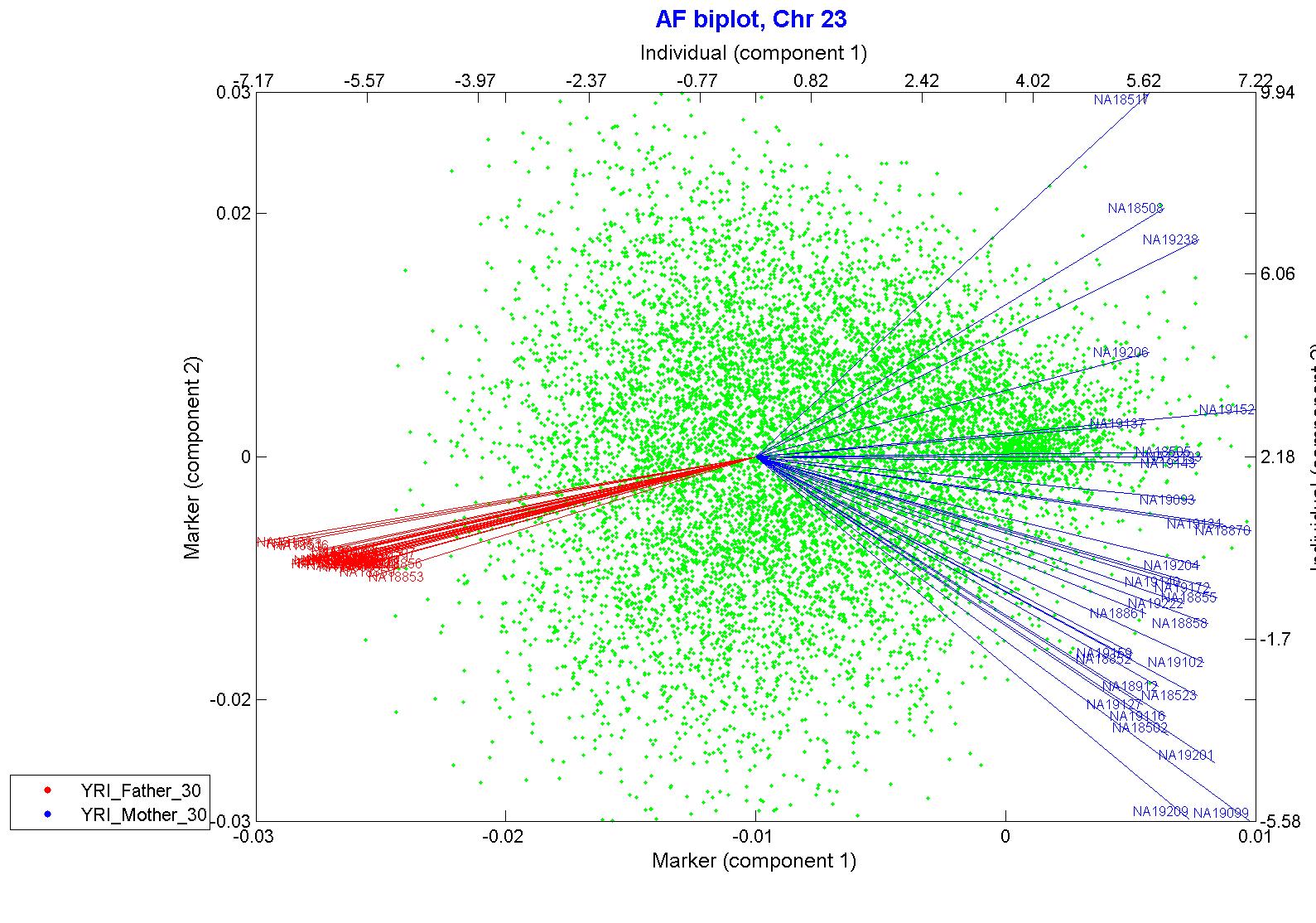
**

**(B)**

**
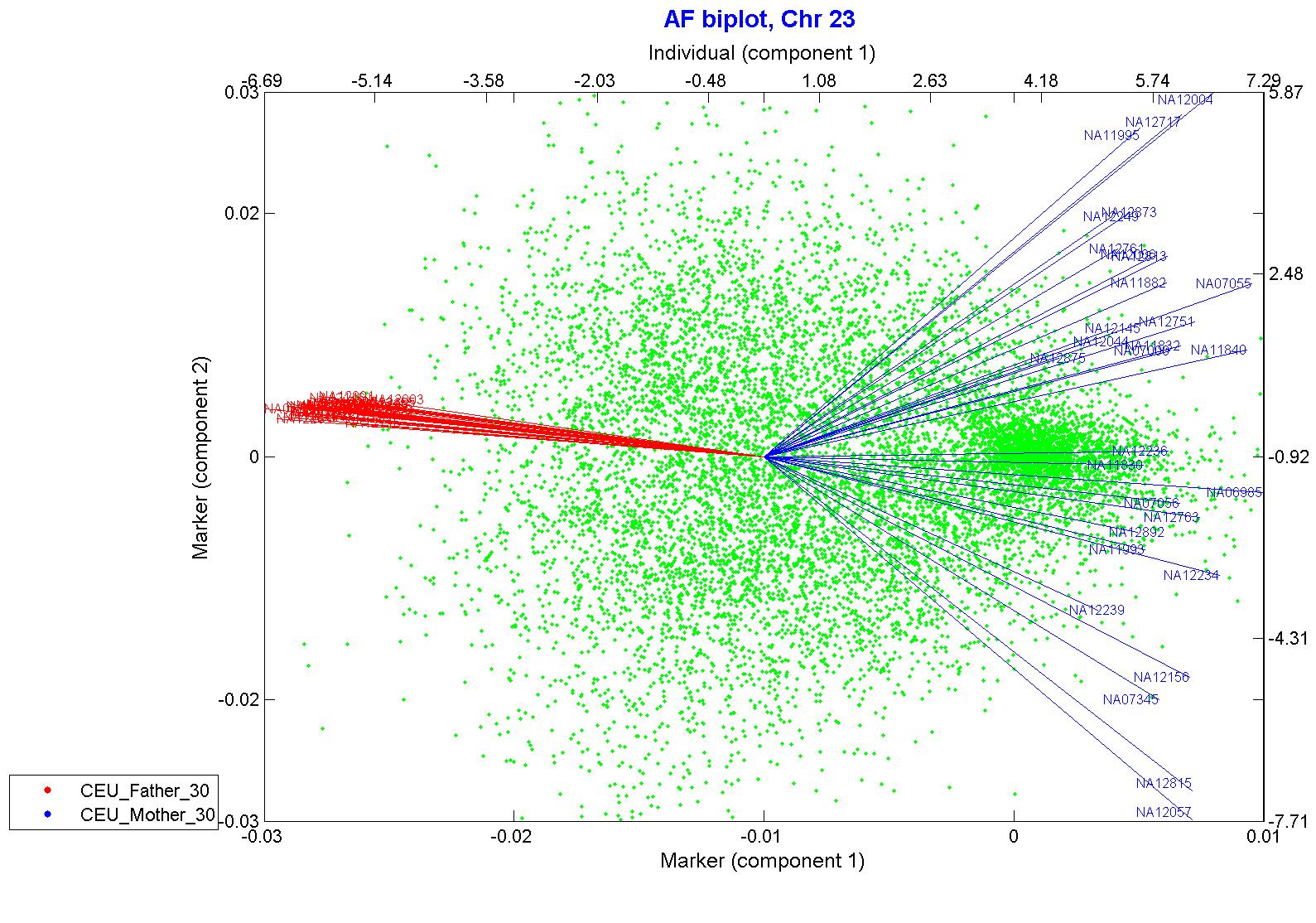
**
