# Supplementary material for: A new analysis tool for individual-level allele frequency for genomic studies
Source: BMC Genomics. 2010 Jul 5;11:415. doi: 10.1186/1471-2164-11-415 (PMC2996943; doi:10.1186/1471-2164-11-415)
Supplement: Additional file 11 — Figure S11.--Allele frequency of a normal sample based on the Illumina HumanHap550-Duo BeadChip. This figure consists of 23 subfigures. Each subfigure presents an allele frequency plot of one chromosome. The vertical axis is the estimated allele frequency, and the horizontal axis is physical position (Mb). Each point denotes a SNP, and the gap in each subplot represents the centromeric gap. The allele frequencies were estimated using an intensity-measuring approach. [file 1471-2164-11-415-S11.DOC]

**Figure S11.**—**Allele frequency of a normal sample based on the Illumina HumanHap550-Duo BeadChip.** This figure consists of 23 subfigures. Each subfigure presents an allele frequency plot of one chromosome. The vertical axis is the estimated allele frequency, and the horizontal axis is physical position (Mb). Each point denotes a SNP, and the gap in each subplot represents the centromeric gap. The allele frequencies were estimated using an intensity-measuring approach.

**
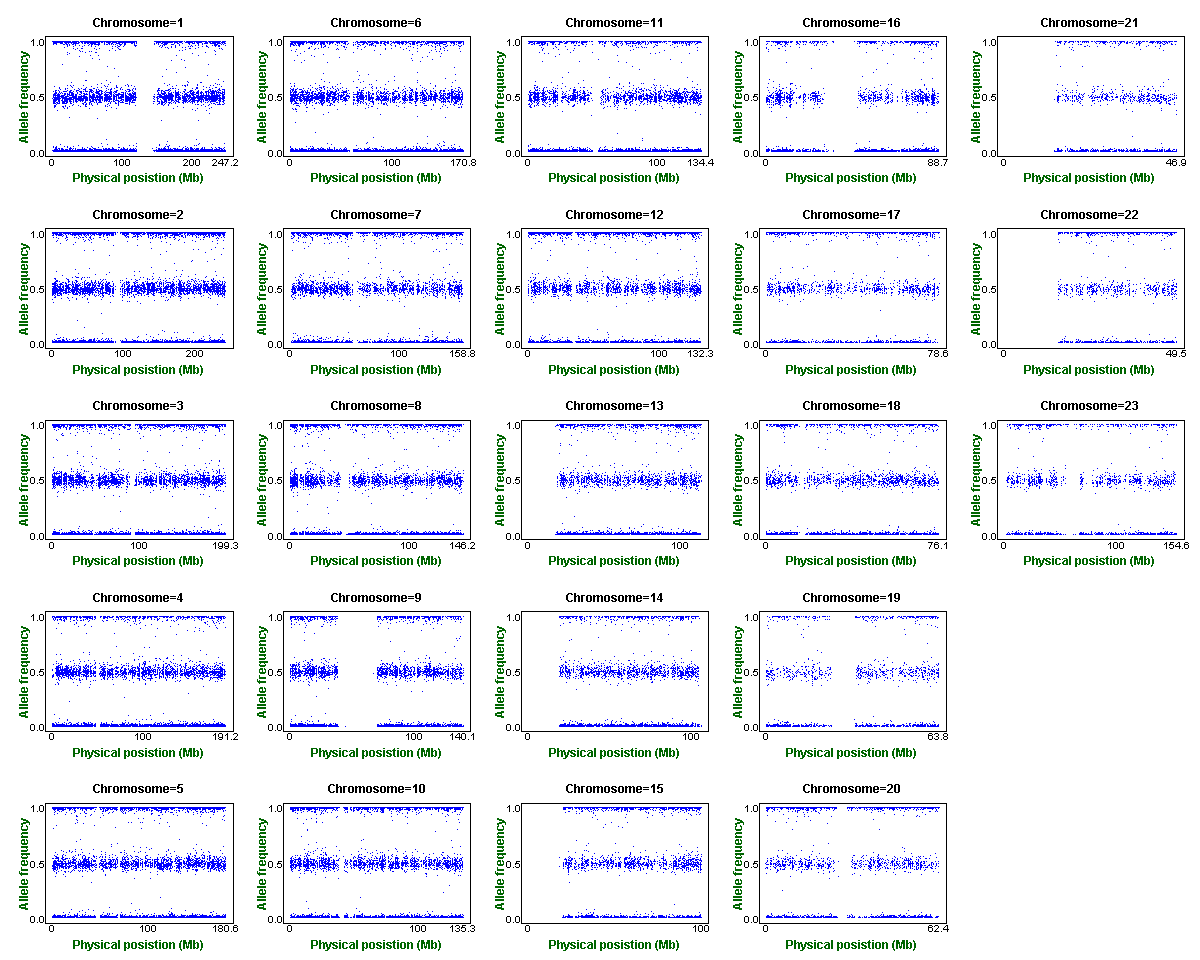
**
